# Perovskites on Ice: An Additive‐Free Approach to Increase the Shelf‐Life of Triple‐Cation Perovskite Precursor Solutions

**DOI:** 10.1002/cssc.202100332

**Published:** 2021-05-28

**Authors:** Mary E. O'Kane, Joel A. Smith, Tarek I. Alanazi, Elena J. Cassella, Onkar Game, Sandra van Meurs, David G. Lidzey

**Affiliations:** ^1^ Department of Physics and Astronomy University of Sheffield Hicks Building, Hounsfield Road Sheffield S3 7RH UK; ^2^ Department of Physics College of Science Northern Border University Arar 73222 (Kingdom of Saudi Arabia; ^3^ Department of Chemistry University of Sheffield Dainton Building, 13 Brook Hill Sheffield S3 7HF UK

**Keywords:** perovskites, photovoltaics, solar cells, solution chemistry, solution processing

## Abstract

The development of stable perovskite precursor solutions is critical if solution‐processable perovskite solar cells (PSCs) are to be practically manufacturable. Ideally, such precursors should combine high solution stability without using chemical additives that might compromise PSC performance. Here, it was shown that the shelf‐life of high‐performing perovskite precursors could be greatly improved by storing solutions at low‐temperature without the need to alter chemical composition. Devices fabricated from solutions stored for 31 days at 4 °C achieved a champion power conversion efficiency (PCE) of 18.6 % (97 % of original PCE). The choice of precursor solvent also impacted solution shelf‐life, with DMSO‐based solutions having enhanced solution stability compared to those including DMF. The compositions of aged precursors were explored using NMR spectroscopy, and films made from these solutions were analysed using X‐ray diffraction. It was concluded that the improvement in precursor solution stability is directly linked to the suppression of an addition‐elimination reaction and the preservation of higher amounts of methylammonium within solution.

## Graphical abstract

**Ice ice baby**: The stability of perovskite precursor solutions is of significant importance as it plays a role in defining the ease by which photovoltaic devices can potentially be manufactured. By studying both solution chemistry and device performance, a simple low‐temperature storage protocol is developed to increase solution shelf life that does not require perovskite composition or stoichiometry to be modified. Devices fabricated from precursor solutions stored at 4 °C for up to one month have the same efficiency as those produced from freshly fabricated controls.

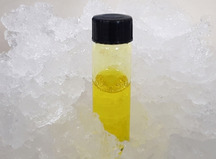

## Introduction

Over the past 10 years, metal‐halide perovskite solar cells (PSC) have achieved impressive single‐junction power conversion efficiencies (PCEs) of up to 25.5 and 29.5 % in tandem architectures with silicon.^[1]^ These perovskites have the general composition ABX_3_, where A is a monovalent cation, typically formamidinium [(CH_3_(NH_2_)_2_^+^, FA^+^], methylammonium (CH_3_NH_3_^+^, MA^+^) or caesium (Cs^+^), B is a divalent metal (such as Pb^2+^ or Sn^2+^) and X is a halide anion (I^−^, Br^−^ or Cl^−^). A significant advantage of this material family is that changes to composition can be used to modify material properties.[^2^, ^3^, ^4^, ^5^, ^6^] For example, the pure formamidinium‐based perovskite FAPbI_3_ can exist as a black photo‐active α‐phase, but is metastable at room temperature and can convert to a photo‐inactive δ‐phase. However, by replacing some fraction of FA with small amounts of Cs^+^ and MA^+^, it is possible to stabilize the black perovskite phase at room temperature.[^5^, ^7^, ^8^, ^9^] Therefore, FA‐rich, triple‐cation (TC), mixed‐halide perovskites compositions have become a popular choice in the research community.

As both the efficiency and operational stability of PSCs has increased, many researchers increasingly look towards the manufacturing requirements of PSCs using roll‐to‐roll processing. Here, solution‐based manufacture is an attractive prospect as it potentially allows PSCs to be produced using low‐cost and high‐throughput techniques. Unfortunately, it has been shown that perovskite precursor solutions are often subject to poor long‐term storage stability[^10^, ^11^, ^12^, ^13^, ^14^, ^15^] (although short‐term aging can improve the performance of resultant PSC devices).[^14^, ^16^, ^17^] This instability presents a significant problem for any practical manufacturing as this will impact on the cost of the process. Indeed, in order to be commercially competitive with vacuum (solvent‐free) deposition techniques, solution‐processing methods need to demonstrate a similar level of process control, despite the additional chemical challenges. To control solution stability, it is clear that an understanding of the interplay between the various organo‐halides, metal halides and solvents in perovskite precursor solutions is essential.

Certain studies on mixed‐cation PSCs have suggested that the poor performance of devices from “aged” solutions result from the degradation of the casting solvents, particularly dimethylformamide (DMF).^[18]^ Indeed, it has been shown that that DMF can hydrolyse into dimethylamine (DMA) and formic acid, especially when accelerated by the presence of lead halides or at high temperature.[^10^, ^19^] The DMA formed can then be protonated by formic acid or another Brønsted acid in solution to form dimethylammonium, [DMA^+^ or (CH_3_)_2_NH_2_^+^],[^19^, ^20^] which can then be incorporated into the perovskite crystal lattice.[^10^, ^18^, ^21^] Similarly, mechanisms have been proposed for DMA^+^ formation in MAPbI_3_ solutions based on the other most commonly used aprotic solvent dimethyl sulfoxide (DMSO).^[22]^

**Scheme 1:**
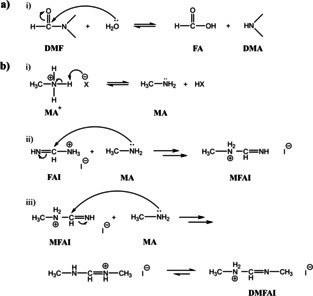
(a) Hydrolysis of DMF to produce DMA. (b) i) Deprotonation of MAX component where X represents a halide ion. ii) Schematic of the addition‐elimination reaction producing MFA. iii) Schematic of the addition‐elimination reaction producing DMFA. For simplicity, the NH_3_ reaction products are not shown. Reaction scheme (b) reproduced from [11].

A different mechanism has however been proposed by Wang et al.^[11]^ to describe the degradation of MA_0.5_FA_0.5_PbI_3_ solutions. Here, it was suggested that the emergence of a non‐perovskite δ‐phase in films prepared from aged solutions occurred as a result of a series of addition‐elimination reactions. In brief, it was proposed that methylammonium is deprotonated to methylamine (CH_3_NH_2_), which can react with FAI to form the condensation product *N*‐methyl formamidinium iodide (MFAI) and, in a secondary process, *N*,*N*‐dimethyl‐FAI (DMFAI), with both reactions releasing NH_3_. Following ref. [11], we summarise both degradation pathways in Scheme 1. In either case, any excess of large organic cations (MFA^+^, DMFA^+^, DMA^+^) in the perovskite is expected to modify the Goldschmidt tolerance factor of the perovskite, forcing the material to adopt a non‐photoactive phase. For large cations, these APbI_3_ phases are generally similar to the aforementioned δ‐FAPbI_3_ (2H polytype) phase, comprising “1D” continuous chains of face‐sharing lead‐iodide octahedra.[^6^, ^23^, ^24^, ^25^] The formation of such cationic species in degraded precursor solutions therefore represents an important process that is expected to prevent the crystallisation of the precursor into a light‐absorbing perovskite phase.

A number of studies have explored various methods to reduce such solution‐aging effects. Kim et al. studied the evolution of FAPbI_3_ perovskite solutions and proposed that the addition of tri‐iodide species can improve the quality of films prepared from aged precursors.^[26]^ Qin et al. have explored the effect of stabilisers^[12]^ on precursor solutions, and showed that the addition of the small molecule ITIC‐Th to FA‐MA‐based solutions can improve solution stability. Wang et al.^[11]^ also found that solution‐aging effects could be controlled through the addition of the stabiliser triethyl borate (TEB) by withdrawing electrons from I^−^, thereby inhibiting the MA deprotonation reaction. Additionally, Shin et al. showed that FAPbI_3_ solutions made from precursor powders are less stable than those made from FAPbI_3_ single crystals using the same solvents,[^13^, ^19^] with Dou et al. demonstrating that solution decomposition can be prevented by instead storing in their precursor state as ball‐milled perovskite powders.^[10]^

Although PSC precursor stabilisers have been identified, it has been shown that even unintentional variations of stoichiometry can dramatically affect device performance.^[27]^ In order to fabricate the highest performing perovskite materials, it is necessary to maintain tight control over the stoichiometry of the precursor solution. Here, the addition of compounds to enhance precursor stability will affect their solution chemistry, and therefore could inadvertently affect perovskite nucleation or charge transport properties within the resultant perovskite films. This in turn may limit device performance or long‐term operational stability. For this reason, the development of methods to enhance solution stability without recourse to molecular stabilisers is seen as a key part of any efficient manufacture process.

In this work, we have examined the effect of long‐term storage on TC perovskite precursor solutions, and using an additive‐free method, have found a way to significantly increase their solution shelf‐life. By studying device performance, the structure of perovskite films fabricated from aged solutions and by directly characterising precursor solutions using nuclear magnetic resonance (NMR) spectroscopy, we identify the formation of both MFA and DMFA in aged perovskite precursors. We show that this reaction results in a significant loss of MA and leads to the formation of non‐perovskite polytypes when such solutions are cast into thin films. We then show that by storing solutions at low temperature, their stability can be significantly enhanced without the necessity to alter solution chemistry. Specifically, we find that devices fabricated using a typical TC precursor solution stored for 1 month at approximately 4 °C achieve a champion efficiency of 18.6 % with a power output of 18.4 % recorded over 1 min. After 2 months storage at approximately 4 °C, such precursor solutions used for PSC devices have >97 % of the champion device performance of freshly prepared solutions and >85 % of this reference device efficiency after 4 months solution storage. Our results also indicate that DMSO‐based precursor solutions are more stable than those made from a DMF/DMSO solvent mixture. Indeed, by using a DMSO‐based precursor, we have fabricated devices using solutions that have been cold‐stored for 4 months, with devices demonstrating a 1 min stabilised PCE of 16.4 %; an efficiency equivalent to 92 % of devices prepared from freshly prepared solutions.

## Results and Discussion

### Device studies

We have prepared several TC precursor solutions, and explored their shelf life under different conditions. These solutions were used both to prepare perovskite films for morphology studies and to fabricate PSC devices. Here, we concentrate on two main solution compositions: a stoichiometric TC solution in DMF/DMSO (here termed *TC‐mixed*) and a stoichiometric TC in DMSO (termed *TC‐DMSO*). Specifically, the composition of the two solutions fabricated was as follows:

- TC‐mixed: TC solution (Cs_0.05_FA_0.81_MA_0.14_PbI_2.55_Br_0.45_) dissolved with a typical 4 : 1 DMF/DMSO solvent volume ratio;
- TC‐DMSO: as TC‐mixed but containing only DMSO solvent.

Here, all stock solutions were stored in amber vials and sealed in a N_2_‐filled glovebox environment. Each PSC device had the basic architecture ITO/SnO_2_/TC‐perovskite/spiro‐OMeTAD/Au [ITO: indium‐doped tin oxide; spiro‐OMeTAD: 2,2′,7,7′‐Tetrakis[*N*,*N*‐di(4‐methoxyphenyl)amino]‐9,9′‐spirobifluorene. The fabrication and testing of perovskite solutions and devices is described in detail in the Experimental Section. For each time point, a series of control devices were fabricated from an equivalent, freshly prepared precursor solution. These controls allowed us to account for any external factors that occurred at each time point that could have affected device performance. Significantly, we find that the control devices show consistent performance over the duration of the experiment when fabricated using the same bottle of solvent (see Figure S2); a result that indicates that the age of the solvent alone does not cause a significant reduction in device efficiency.

To understand the aging of the TC‐mixed precursor solution (Cs_0.05_FA_0.81_MA_0.14_PbI_2.55_Br_0.45_), we first explored the effect of different storage temperatures on device performance. Solutions were stored at 4, 22, 45 and 70 °C over a period of 17 days. The metrics of devices prepared from the solutions are shown as a function of solution aging time in Figure S3. Here, devices prepared using solutions stored at 70 °C for 17 days have deteriorated greatly in performance, with their efficiency reduced to around 30 % of the control devices prepared from a freshly made precursor solution. Notably, devices fabricated from solutions stored at 4 °C had efficiencies consistent with the experimental controls. We have used this experiment to calculate an effective activation energy for the degradation process of 0.31 eV using the Arrhenius relationship^[28]^ (shown in Figure S3). Although, this energy is an order of magnitude larger than room temperature, it is not inconsistent with that determined in other processes that occur over a period of days or weeks at room temperature.[^28^, ^29^]

**Figure 1:**
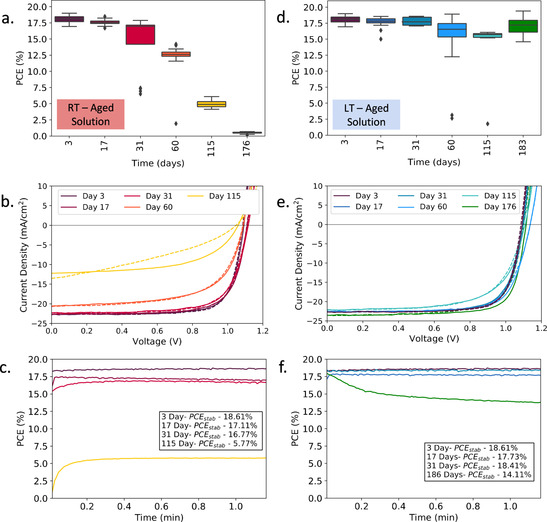
(a) PCE of perovskite device with the stack ITO/SnO_2_/perovskite/spiro‐OMeTAD/Au fabricated from RT‐aged TC‐mixed (DMF/DMSO) solutions. (b) *J*‐*V* curves and (c) stabilized power output for the best performing devices from RT‐aged solutions. (d) PCE of devices fabricated from low‐temperature‐aged solutions, with (e) *J*‐*V* curves and (f) stabilized power output for the best performing cells. Full *J*‐*V* sweep parameters for (b) and (e) and all device data for boxplots in (a) and (d) are given in Tables S2 and S3.

These measurements indicate that storage of the precursor solution at lower temperature increases their useful lifetime. We therefore performed an extended study in which we compared devices fabricated from TC‐mixed and TC‐DMSO precursor solutions that were either stored in a glovebox at room temperature (≈22 °C) or refrigerated (at ≈4 °C). These conditions will henceforth be referred to as RT‐ and LT‐aged, respectively. Figure 1a shows the PCE of devices fabricated from the RT‐aged TC‐mixed precursor as a function of storage time. The average and best PCE of devices fabricated from the freshly prepared solution was (18.1±0.5) and 19.0 %, respectively. There is a noticeable reduction in the efficiency of devices prepared from solutions stored for approximately 30 days at RT. Devices prepared from solutions stored for four months have an average PCE of (5.0±0.6)% (with 6.1 % best sweep). This effect primarily results from a reduction in fill factor (FF) and short‐circuit current (*J*_SC_) (see Figure S4). Notably, devices prepared from solutions stored for 186 days (6 months) demonstrated no photovoltaic response. For completeness, we plot the *J*‐*V* sweeps for the best performing devices fabricated at each time‐point during the storage experiment in Figure 1b (see full device metrics in Tables S2 and S3). Here, a significant hysteresis in the *J*‐*V* characteristics is observed for devices fabricated from solutions stored for >2 months. The stabilised power output for these devices is shown in Figure 1c, where a reduction in PCE is evident. As can be seen in Figure S5, we observed similar trends with precursor aging for devices prepared from TC‐DMSO solutions; however, when DMSO was used as a primary solvent, we find that the resultant devices had improved performance compared to those prepared from DMF/DMSO precursor that had been aged over 31 and 186 days (see Tables S4 and S5).

We now discuss the performance of devices fabricated from precursor solutions stored at 4 °C. Figure 1d shows device PCE as a function of storage time for the LT‐aged TC‐mixed solution, with *J*‐*V* curves and power output for the best devices recorded over a period of 1 min plotted in Figure 1e,f, respectively. Here, Figure 1d confirms that over a 1‐month storage period, there was negligible degradation in device performance compared to RT‐aged solutions. Specifically, after 31 days solution‐aging, the devices fabricated had an average and best efficiency of (17.8±0.6) and 18.6 %, around 97 % of the PCEs achieved by devices made from fresh solution. This enhanced stability is reflected in device stabilised power output; here “champion” devices prepared from solutions stored for 3 days and 1 month had stabilised efficiencies of 18.6 and 18.4 %, respectively.

We find that devices fabricated from solutions that have been cold‐stored for 2 months have some degree of reduced performance; however, this effect is less significant than for those fabricated from RT‐aged solutions. Devices fabricated from cold‐stored solutions for 4 months had efficiencies that were >85 % of those prepared from freshly made solutions, with average and best PCEs of (15.7±0.3) and 16.0 %. Furthermore, after 6 months of cold solution‐storage, devices had impressive initial sweeps. It can be seen however that there is a rapid (30 s) burn‐in (loss of efficiency) seen in the device made from the 6 month LT‐aged solutions (see Figure 1f), with the device efficiency not stabilising completely over the course of the measurement. Nevertheless, the champion device made from this solution had a PCE of 14.1 % after 1 min. We suspect that even though low‐temperature storage significantly slows reaction rates, there is a gradual build‐up of degradation products in solution that is likely to have a negative impact on the long‐term operational stability of the finished devices. More experiments are planned to explore such effects.

Interestingly, we find a slightly higher level of stability from TC‐DMSO solutions stored at 4 °C compared to those based on the mixed solvent (see Figure S6). Indeed, although devices fabricated from the TC‐DMSO solution had a lower initial performance [(16.6±1.7) and 18.0 % best efficiency], this solution achieved (16.7±0.6)% after 4 months of LT storage. The champion device fabricated at this 115 day time point had an initial sweep of 17.5 % with a stabilised efficiency of 16.4 % This preliminary result suggests that precursor solutions prepared from DMSO may have better intrinsic stability than those based on DMF.

### Film morphology

**Figure 2:**
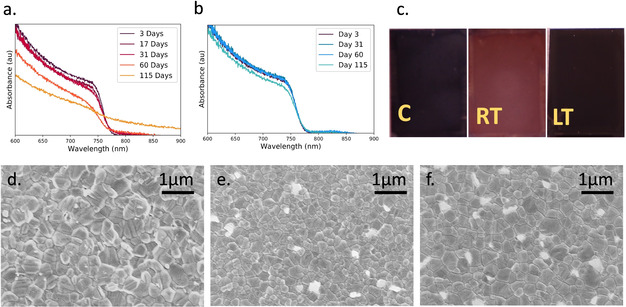
UV/Vis absorption spectra of TC‐mixed perovskite films prepared from solutions that have been aged at (a) RT and (b) at a low temperature of 4 °C. (c) Images of films prepared from a precursor solution aged for 115 days at RT and at 4 °C (LT) next to a film fabricated from a freshly prepared control ink (marked C). SEM images comparing films fabricated from (d) RT‐ and (e) LT‐aged (115 days) solutions to those fabricated from a (f) freshly prepared control solution.

To explore the origin of the observed changes in device efficiency, we have investigated the optical and structural properties of perovskite films prepared from freshly prepared (control) and aged solutions. Here, Figure 2a,b plots the UV/Vis absorption of films made from TC‐mixed solutions stored at RT and LT, respectively. It is immediately apparent that films prepared from solutions stored at RT undergo a progressive reduction in optical density. Indeed, the film prepared from the solution stored at RT for 115 days (≈4 months) has no clear absorption onset, with an absorption tail that extends to long wavelengths being characteristic of a significant degree of optical scattering. The absorption spectra for films prepared from the LT‐aged solution appear much more self‐similar; here, a small reduction in optical density is only apparent in the film prepared from the 115‐day stored solution.

These observations are confirmed in Figure 2c (and Figure S7) where we present images of films prepared from a control solution and solutions stored for 115 days at RT or LT. Here, the film prepared from the control solution is black and specular, whereas the film prepared from the solution aged at RT appears cloudy and less optically dense, indicating a rougher film. This roughness is consistent with the increased optical scattering observed in absorption measurements shown in Figure 2a. This is further supported by atomic force microscopy (AFM) shown in Figure S8, confirming an increase in roughness for films produced from RT‐aged precursor solutions. Here, the root mean square (RMS) roughness determined from freshly prepared (control) solutions and solutions aged at LT and RT is 26, 30 and 80 nm, respectively. We also observe similar effects for DMSO‐based solutions aged at RT (see Figure S9).

Remarkably, in contrast to the RT‐aged film, the film prepared from the solution stored at LT is consistent in appearance with the control film and has formed a photoactive perovskite phase. To investigate this effect, we used scanning electron microscopy (SEM). Such grains have a “striped” appearance; an effect consistent with crystallographic twinning.[^30^, ^31^] Twinning effects generally result from a local increase in strain during film formation, likely resulting from a lattice mismatch within the crystal. Note that while bright grain boundaries are suggestive of inorganic‐rich grain boundaries, we cannot exclude the possibility that such effects result from inefficient charge transfer between the grains. It is apparent, however, that no significant changes in film structure are observed in samples prepared from precursor solutions stored at LT.

### Crystal structure

**Figure 3:**
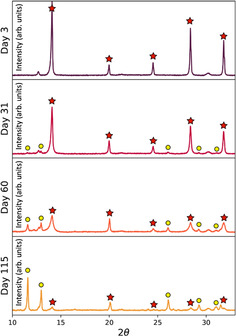
XRD patterns for films made from RT‐aged TC‐mixed solutions after 3, 31, 60 and 115 days. Red stars mark cubic perovskite peaks and yellow circles mark emerging intermediate peaks.

X‐ray diffraction (XRD) was used to explore the crystal structure in films produced from control and aged precursor solutions. Figure 3 plots XRD data recorded from films fabricated from the TC‐mixed solution aged at RT for 3, 31, 60 and 115 days. Here, peaks consistent with a pseudo‐cubic TC perovskite are labelled with red stars, while yellow circles identify new peaks (comparative data recorded from TC‐DMSO cast films are shown in Figure S10). Figure 3 shows there is a significant solution age‐dependent reduction in scattering from the perovskite 100 reflection at 2*θ*=14.1°. This process is accompanied by an increase in scattering from new peaks at 11.6°, along with peaks at 12.9, 26.0, 29.2 and 31.5°. This reduction in scattering intensity from the 14.1° peak and the emergence of a peak at 11.6° with those between 25–33° coincide with those expected for a 4H hexagonal polytype (see Figure S12). The TC‐DMSO sample also exhibits reflections consistent with 6H phases being present in the film. Our measurements indicate that these polytypic phases preferentially form in films prepared from RT‐aged solutions, with the formation of various polytypes being dependent on the casting solvent, and in turn the degradation of the organo‐halide component of the solution.

**Figure 4:**
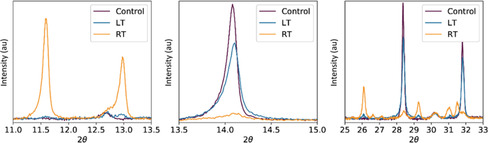
XRD highlights for films made from TC‐mixed solutions that have been stored for 115 days at RT (yellow) and LT (blue) together with a freshly prepared control film (purple). Here, films from the LT‐aged ink still exhibit strong scattering from the perovskite phase, whereas in the RT‐aged solution a non‐perovskite phase is present (see Figure S12).

Figure 4 compares XRD scattering patterns from films made with a control TC‐mixed precursor solution, with films prepared from solutions aged for 115 days at RT or LT. It can be seen that the reduction of the tetragonal perovskite phase (evidenced by the peak at 14.1°) and the growth of the peaks at 11.6, 12.9 and 26.0° are strongly suppressed when solutions are stored at 4 °C; a result consistent with the thin‐film absorption and SEM measurements presented in Figure 2 and the device studies shown in Figure 1. This same effect is seen for the TC‐DMSO solutions stored at low temperatures (Figure S13). Our results suggest therefore that storage at low temperature strongly suppresses the formation of undesirable polytype phases.

### NMR solution study

**Figure 5:**
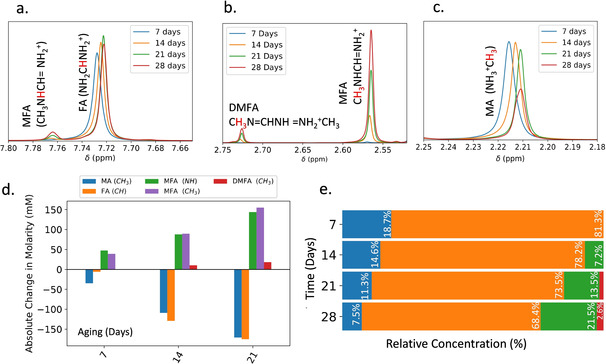
(a–c) NMR spectra of TC‐mixed solution dissolved in DMF/DMSO measured at several time points over a 1‐month period (full spectra in Figures S15–S18). (d) Absolute change in molarity for the various organic components over time, estimated compared to an internal standard for which the molarity remains constant. (e) Relative concentration of the organic cations over time determined as described in Supporting Information Note 3. Here we see a significant reduction in the relative concentration of both MA and FA, along with an increased concentration of MFA and DMFA.

We have used NMR to analyse the composition of TC solutions as they are aged in d_7_‐DMF, d_6_‐DMSO or a d_7_‐DMF/d_6_‐DMSO mix. Figure 5 shows NMR spectra recorded from a TC precursor dissolved in 4 : 1 d_7_‐DMF/d_6_‐DMSO, measured weekly over 28 days (full spectra for all solutions can be found in Figures S15–S24) and subsequent analysis. Here, we assign the peaks at 2.2 and 7.7 ppm to the CH_3_ and CH protons in MA and FA, respectively. We find a different chemical shift for these peaks in the different solvents (DMF vs. DMSO), which we suspect results from solvent interaction effects.[^32^, ^33^] We discuss this effect further in the Supporting Information. Figure 5a,b shows the formation of three new peaks around 2.57, 2.73 and 7.77 ppm as solutions age. As shown in Figure 5c, this growth is correlated with a significant decrease in intensity of the MA CH_3_ peak (2.20 ppm) after 28 days aging. On the basis of previous work by Wang et al.,^[11]^ we assign the peaks at 2.57 and 7.79 ppm to the methyl and amine groups of MFA, respectively, with the peak at 2.74 ppm ascribed to the methyl group of DMFA. We note however that previous work has described the hydrolysis of DMF to form DMA, and its subsequent protonation to produce DMA^+^. DMA^+^ has an ^1^H NMR peak at approximately 2.55 ppm^[34]^ (i. e., overlapping with the peak that we assign to the methyl group of MFA). To determine whether DMA does in fact contribute to our measured NMR spectra, we have integrated the area under the peaks at 2.57 and 7.79 ppm and find them to have an area ratio of (3.0±0.3):1. This intensity ratio is consistent with the 3 : 1 proton ratio expected for the CH_3_ and NH groups in MFA, and thus we conclude that our solutions do not contain a substantial concentration of DMA^+^. We can gain further confidence in this conclusion by performing ^2^H (deuterium) NMR on precursors (see Figure S14). This measurement probes the relative composition of the deuterated solvents and is insensitive to changes in the molecular cations. Figure S14 shows NMR spectra for the deuterated DMSO and DMF/DMSO precursor solutions both before and after 6 months storage at RT. Here, no changes are observed in the deuterated spectra after this time period; a result that indicates that any degradation in the precursor solutions does not result from deterioration or chemical changes to the solvent.

We can use our ^1^H NMR spectra to gain a quantitative understanding of changes in precursor chemistry. Here, we have used an internal standard of known concentration in an isolated capillary tube (seen at 8.436 ppm) to determine absolute molarity changes of MA, FA, MFA and DMFA in solution as a function of time (full method described in the Supporting Information).

Absolute molarities of organic component within the TC solution in DMF/DMSO are shown in Figure 5d with NMR spectra shown in Figures S15–S18. We find that over a period of 29 days, the quantity of FA and MA both reduce by a similar concentration (170 mm); a finding that may indicate that these components react with each other. Over the same time period, the increase in the integrated area under the peaks at 2.57 and 7.79 ppm indicate the formation of approximately 150 mm of MFA, with the peak at 2.74 ppm indicating the formation of approximately 20 mm of DMFA. The total concentration of MFA and DMFA formed over this time period is thus approximately 170 mm; a value comparable to the molarity loss of MA and FA. This simple analysis confirms the reaction proposed by Wang et al.,^[11]^ and suggests that the main degradation pathway in high‐efficiency perovskite precursor solutions involves the deprotonation of methylammonium to methylamine initiating an addition‐elimination reaction, thus forming the condensation products MFA and subsequently DMFA.

We have applied the same methodology to explore changes in the chemistry of precursor solutions utilising a DMSO solvent. Here, NMR spectra recorded from DMSO‐only precursor solutions are shown in Figure S25. Our analysis indicates that a combined total of approximately 65 mm MFA and DMFA form in the DMSO‐only precursor solution after 28 days. This again confirms that the solvent has an impact on the rate of chemical decomposition.

**Figure 6:**
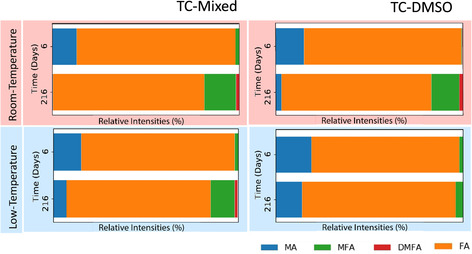
Relative intensity of different cationic species in the TC precursor solutions after 6 and 216 days after storing at either RT or 4 °C (LT). Data is shown for solutions prepared from a DMF/DMSO solvent mix or DMSO‐only.

Finally, we have used NMR to examine the effect of storage temperature on the precursor solution degradation process. Figure 6 shows the relative intensities of peaks characteristic of MA, MFA, DMFA and FA in either a DMF or DMF/DMSO solvent before and after 6 months (216 days) storage in LT and RT conditions. Here, the relative composition was calculated using the same method as was used in Figure 5. In this experiment, however, no internal standard was present, and absolute concentrations cannot be calculated for each species. However, we have referenced the relative intensities of each of the peaks to a residual DMSO peak present in all samples, whose intensity was not found to change over the course of the experiment. As it can be seen, we find that a similar amount of MFA and DMFA are formed in DMF/DMSO based solutions regardless of storage conditions. In all cases however, we note a reduction in the relative concentration of MA in the aged solutions, with this effect being most significant in the RT‐aged DMF/DMSO TC solution. Indeed, the complete absence of MA in solution correlates with our device studies in which we demonstrated that this precursor solution could not be used to create a perovskite film after storage for a similar time period at RT. It is also apparent that the formation of MFA and DMFA in the DMSO‐based precursor solution is significantly suppressed when it is stored at LT. This finding further supports our conclusion that TC perovskites are generally more stable in DMSO‐based solutions.

## Conclusions

We have studied the aging of high‐performance triple‐cation (TC), mixed‐halide precursor solutions and have found that such effects are strongly dependent on temperature. Using NMR spectroscopy, we have identified the primary mechanism of degradation in these solutions to be the deprotonation of methylammonium, and the subsequent formation of *N*‐methyl formamidinium (MFA) and *N*,*N*‐dimethyl formamidinium (DMFA). This change in the proportion of organic components will directly alter the tolerance factor of the perovskites and drive the formation of non‐perovskite polytype phases. This is confirmed using X‐ray diffraction, which shows that films cast from RT‐aged solutions contain a greater fraction of non‐perovskite phases, resulting in devices having significantly reduced performance. Furthermore, it appears that the choice of precursor solvent affects the decomposition to larger cations and consequently the polytypes that form.

With low‐temperature solution storage, this process can be significantly suppressed without altering the solution chemistry. Specifically, we show that devices that have been fabricated from a TC perovskite dissolved in DMF/DMSO can be stored for 1 month at 4 °C with negligible deterioration in device performance. Indeed, using this approach we show that even after 4 months cold storage, such solutions can be used to fabricate devices with a power conversion efficiency (PCE) of (15.7±0.3)% and retain >85 % of the efficiency of devices prepared from freshly prepared solutions.

We find that solvent choice has a significant impact on solution stability. For the particular TC perovskite studied, we find DMSO can be used to create a more stable precursor solution than the widely used DMF/DMSO solvent mixture. Although devices prepared from this precursor have lower initial efficiencies, we find that devices fabricated from DMSO‐only precursor solutions are more stable; for example, they can be stored for 4 months at 4 °C and can then be used to create devices having a champion PCE of 17.1 % with a stabilised efficiency of 16.4 %, a value that is >90 % of devices made from freshly prepared solutions. Previous work has indicated that solvent reactions involving DMSO require a significantly higher temperature^[35]^ than those that occur in DMF‐based solvents.[^10^, ^18^, ^19^] We speculate that the observed increased stability may stem from the higher basicity of DMSO. DMSO may establish more hydrogen bonding with methylammonium in solution, supressing the aforementioned addition‐elimination reaction. It is also possible the solvent degradation products (even in small amounts) may drive reaction kinetics forwards, however we note that our NMR data shows that no significant amount of these products (namely dimethylamine) are formed during aging. More generally, our results demonstrate that with correct storage conditions, solution stability can be maintained for a significant period without the need to alter solution chemistry. We believe that future work should address the long‐term operational lifetime of devices made from aged solutions and also explore the impact of intrinsic factors (such as solvent basicity) on solution stability.

## Experimental Section

### Precursor solution preparation

All solvents were purchased from Sigma Aldrich. PbI_2_ and PbBr_2_ were purchased from TCI Chemicals, MAI and FABr were purchased from Ossila, and Caesium Iodide was purchased from Sigma Aldrich. The stoichiometric inks prepared contained 467.5 mg mL^−1^ of PbI_2_, 167.2 mg mL^−1^ of MAI, 68.3 mg mL^−1^ of PbBr_2_ and 18.8 mg mL^−1^ of MABr. These were weighed out and dissolved in each of the relevant solvents (DMF/DMSO or DMSO). A stock solution of CsI was prepared in DMSO (389 mg mL^−1^), with 40 μL of this solution then added to the solution. The final stoichiometry of the resultant solutions was Cs_0.05_FA_0.81_MA_0.14_PbI_2.55_Br_0.45_.

### Device fabrication

Devices were fabricated on ITO coated glass that was patterned via etching using zinc powder and 4 m HCl. Substrates were cleaned using a surfactant (Hellmanex) solution to remove excess zinc and then sonicated in a Hellmanex solution, deionised (DI) water and isopropyl alcohol (IPA) for 20 min, 5 min and 15 min, respectively.

PSC devices had the following device structure: glass/ITO/SnO_2_/perovskite/spiro‐OMeTAD/Au. To fabricate devices, substrates were first placed in a UV‐ozone cleaner for 20 min. A suspension of 15 % SnO_2_ nanoparticles in H_2_O was diluted to 4 : 1 with H_2_O. 50 μL of this solution was then spin coated at 3000 rpm for 30s onto the ITO substrate. These samples were then exposed to a UV‐ozone (for 20 min) before being transferred to a nitrogen‐filled glovebox. Perovskite layers were then deposited from the precursor solutions via spin coating, with the spin‐coating parameters listed in Supplementary Note 1. All perovskites were “quenched” during spin‐coating using 100 μL of an ethyl acetate antisolvent to promote crystal nucleation.

A spiro‐OMeTAD hole transport layer (85 mg mL^−1^ in chlorobenzene) was spun onto the perovskite. The following dopants were added to this solution: 34 μL mL^−1^ TBP, 11 μL mL^−1^ FK209 in ACN (300 mg mL^−1^ concentration) and 20 μL mL^−1^ of LiTFSI in ACN (500 mg mL^−1^). This solution was filtered directly before use, and was spin coated by dynamically dispensing 25 μL of solution at 4000 rpm and coating for 30 s. The coated substrates were stored in dry air overnight and then placed in an Edwards Auto 306 bell‐jar evaporator. A gold electrode layer (80 nm) was then evaporated onto the substrates through a shadow‐mask to create 6 pixels with each pixel having an area of 0.02506 cm^2^.

Devices were tested using a Newport 92251A‐1000 solar simulator in ambient conditions, with *J*–*V* measurements recorded using a Keithley 237 source measure unit. Measurements were made by reverse sweeping the bias between −0.1 and 1.2 V at 100 mV s^−1^. Device stabilised power output was measured by holding devices for several minutes at a bias voltage defined by the reverse sweep *V*_mpp_. All device metrics were typically measured one day after perovskite deposition.

### Absorption and thicknesses

UV/Vis measurements were performed under ambient conditions using UV/VIS/NIR light source (Ocean Optics – DH‐2000‐BAL), collection fibre optic cables (Ocean Optics) and spectrometer (Ocean Optics – HR2000+ES). Samples for absorption measurements were prepared on quartz‐coated glass using the same deposition methods as used in device fabrication. Thickness measurements were performed using a DekTak XT surface profilometer to scan over a scratch made in the film. Measurements were made at several places across each film, and an average and standard deviation then determined.

### SEM, AFM and XRD

Samples for SEM, AFM and XRD were prepared on ITO coated glass that had been coated with SnO_2_ and perovskite films using the same deposition parameters as used to create devices. AFM images were taken using Bruker Dimensions 3100 in Intermittent Contact Mode using BrukerTESPA‐V2 cantilevers with a spring constant of 42 N m^−1^. Roughness measurements were taken using Gwyddion image analysis software. SEM imaging was performed using an FEG‐Raith SEM operating at a beam energy of 1.5 kV at a working distance of 4–5 mm, with an in‐lens detector used to collect backscattered electrons. XRD measurements were performed using a PANalytical X'Pert Pro system. This was equipped with Copper Line Focus X‐ray tube run at a voltage of 45 kV with a tube current of 40 mA, with data collected using a 1D‐detector, in Bragg‐Brentano geometry.

### NMR spectroscopy

For solution‐NMR measurements, samples were prepared using deuterated solvents sourced from Sigma Aldrich. Peak positions were calibrated with respect to an internal isolated standard of 1,2,4,5‐tetrachloro‐3‐nitrobenzene (TCNB) at 8.4364 ppm in DMSO‐d6 in a capillary tube. ^1^H NMR and ^2^H measurements were performed using a Bruker AVANCE III instrument operating at 400.2 MHz and 61.43 Hz, respectively, at 289 K. Spectra were recorded in 16 scans using over a 20 ppm spectral width with 64k acquisition points and a relaxation delay of 30 s to ensure full quantification. All solutions were sealed into 100 MHz, 7“ Standard Series NMR tubes in a nitrogen‐filled glovebox. Molarity changes as shown in Figure 5 were estimated using TopSpin software and referenced to the TCNB internal standard that had a molarity of 383 mm. This is further explained in the Supporting Information. The measurements presented Figure 6 were locked onto a DMSO‐d6 solvent peak.

## Conflicts of interest

David Lidzey is a director and shareholder of the materials science company Ossila Ltd that retails equipment and materials (including perovskite inks) for photovoltaic device research.

## Supporting information

As a service to our authors and readers, this journal provides supporting information supplied by the authors. Such materials are peer reviewed and may be re‐organized for online delivery, but are not copy‐edited or typeset. Technical support issues arising from supporting information (other than missing files) should be addressed to the authors.

Supplementary
