# Supplementary material for: Perovskites on Ice: An Additive‐Free Approach to Increase the Shelf‐Life of Triple‐Cation Perovskite Precursor Solutions
Source: ChemSusChem. 2021 May 28;14(12):2537–46. doi: 10.1002/cssc.202100332 (PMC8251910; doi:10.1002/cssc.202100332)
Supplement: Supplementary file 1 — Supplementary [file CSSC-14-2537-s001.pdf]

ChemSusChem

Supporting Information

Perovskites on Ice: An Additive-Free Approach to Increase the Shelf-Life of Triple-Cation Perovskite Precursor Solutions

Mary E. O'Kane,* Joel A. Smith, Tarek I. Alanazi, Elena J. Cassella, Onkar Game, Sandra van Meurs, and David G. Lidzey*© 2021 The Authors. ChemSusChem published by Wiley-VCH GmbH. This is an open access article under the terms of the Creative Commons Attribution License, which permits use, distribution and reproduction in any medium, provided the original work is properly cited.

Experimental Methods

Note 1 - Device Fabrication.

Table 1 shows the optimised process recipes used for both precursor solutions.

Solution	Spin Cycle	Quenching Time	Annealing temp	Annealing time
TC-mixed	Acceleration<br>200rpm/s for 5s.<br>1000rpm/s for 1s<br>2000rpm for 10s<br>4000rpm for 20s	10s into last step,<br>10s before end.	100°C	60 min
TC-DMSO	2000rpm for 10s<br>4000rpm for 28s	13s into last step,<br>15s before end	130°C	10 min

Table 1. Spin coating parameters for PSCs/films described in the study.

Note 2 - Arrhenius Relationship

We explored the time-dependent aging of the perovskite precursor solutions at a series of different temperatures. This allowed us to estimate an overall activation energy for the reactions taking place. Here, we used the Arrhenius relationship[1] which we have used to relate the reduction in device efficiency (caused by the degradation of the precursor solution) to its storage temperature.

$$\ln(\lambda) = -\frac{E_A}{k_B T} + \ln(A) \quad (1)$$

Here, E_A is an activation energy, k_B is the Boltzmann constant, T is the solution storage temperature, A is a fitting constant and λ is the degradation rate. Here, we define degradation rate using the following equation

$$\lambda = \frac{1-\left({PCE_n}/{PCE_{Initial}}\right)}{n} \quad (2)$$

where $PCE_{Initial}$ is the average initial PCE of devices made from freshly prepared solutions and PCE_n is the average PCE for devices that had been prepared from solutions that had been aged for *n* hours.

Note 3 - NMR Analysis

All analysis were carried out using TopSpin software. MA and FA peaks were identified by their chemical shift and comparison to ^{1}H reference spectra of individual components. To estimate absolute molarity changes, the area under each peak in the aged precursor solution was integrated and compared with the area under the internal standard. Overlapping peaks were deconvoluted using TopSpin software to extract their individual intensities. The internal standard used was 1,2,4,5-tetrachloro-3-nitrobenzene which was prepared at a concentration of 383 mM in d_6-DMSO. This peak can be seen at 8.436 ppm in Fig S14-S23. This calibration peak however overlaps with a broad peak at ~8.4 ppm. We speculate this

broad peak is made from several unresolved peaks corresponding to the NH components of FA and MA usually found at 9.0 ppm and 8.7 ppm for FA and 7.8p pm for MA. Here, we suspect that significant proton exchange between molecules may cause these peaks to merge and broaden. To isolate the signal corresponding to the TCNB standard (and thereby also determine the area of the other peaks), we have use the deconvolution feature in the TopSpin software. An example of this deconvolution process is shown in Figure S1a. Here it can be seen that the individual peaks can be successfully isolated and their integrated area determined. We acknowledge that this deconvolution process may lead to an uncertainty in the integration values which we believe to be around ±10%.

We have also performed a ^{1}H NMR calibration experiment, measuring TC solutions containing 3 different total solids concentrations: 225mg/ml, 774mg/ml and 901mg/ml. For each of these samples, the integrated areas of the FA-CH and MA-CH_3 peaks are calibrated with reference to the area of the (known concentration) TCNB standard, with this process utilised throughout this study. We present the results of this experiment in Figure S1b. Figure S1b show that the molarities determined scale in an approximately linear fashion with concentration for both the MA and FA peak; a result that indicates that this methodology can accurately measure concentration within solution.

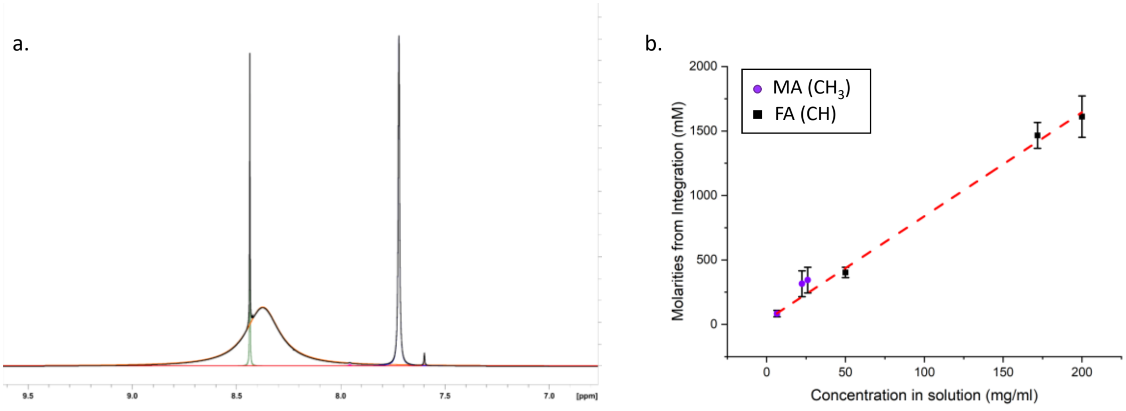

Figure S1a. *Part of NMR spectra for TC-DMF-DMSO sample showing deconvoluted peaks and with the combined line shape (black).* ***b.*** *Concentration calibration experiment conducted. Here, the molarities calculated from the deconvoluted MA-CH_3 peak (purple circles) and FA-CH peak (black squares) are plotted against measured sample concentrations for different sample concentration. As expected, there is a linear relationship between sample concentration and integral area (see the linear fit in red).*

The molarity determined from each peak is detailed in Tables 6-8. The measured intensities have been scaled where necessary to calculate molarity to compensate for the number of hydrogen atoms (or protons) contributing to it, e.g. in each MA CH_3 group there are three hydrogen atoms which contribute to one peak. Absolute molarity changes shown in Figure 5d and SW were calculated using $\Delta M = M_{init} - M_P$, where ΔM is the change between the initial molarity M_{init} and the molarity at time point, M_P. The relative (*RM*) molarity (expressed as a %), (as shown in Figure 5e) is calculated by normalising individual molarities, M_x, to the total molarity of the A-cations using

$$\text{RM (\%)} = \frac{M_x}{M_{MA}+M_{FA}+M_{MFA}+M_{DMFA}} \qquad (3)$$

Supplementary Figures

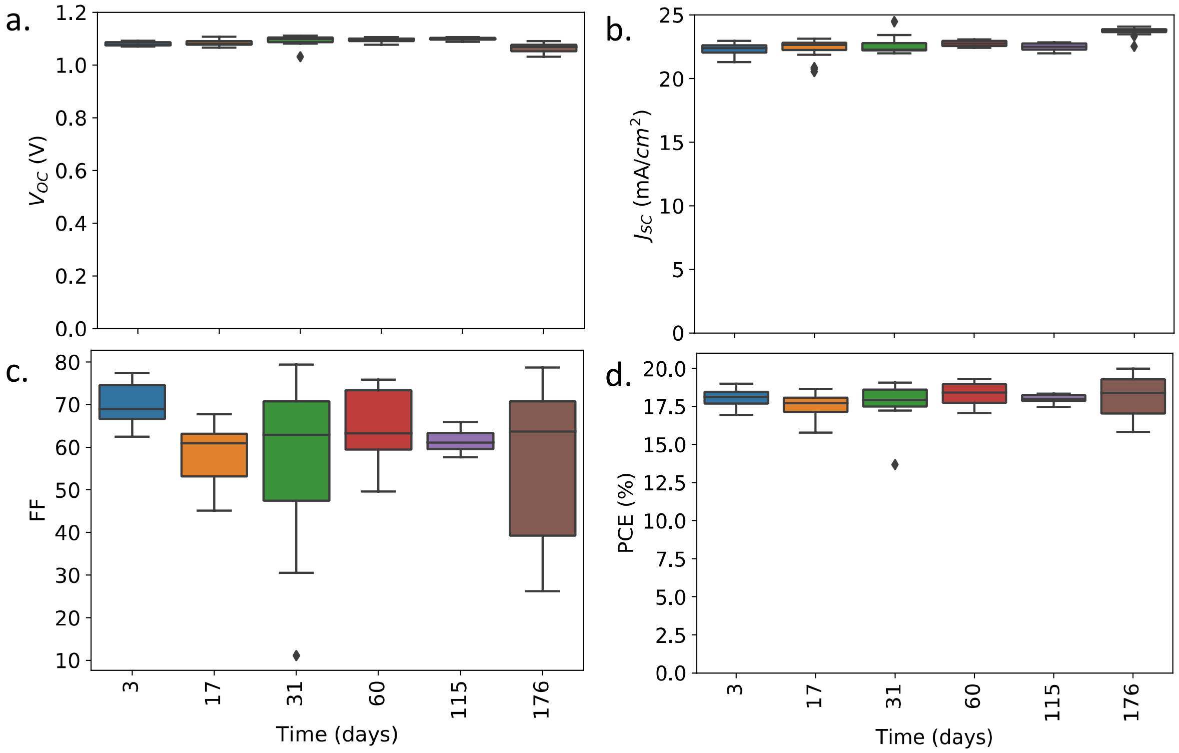

Figure S2. *Boxplots showing V_{OC} (a), J_{SC} (b), FF (c) and PCE (d) for control devices made from a fresh TC-mixed solution at each time point.*

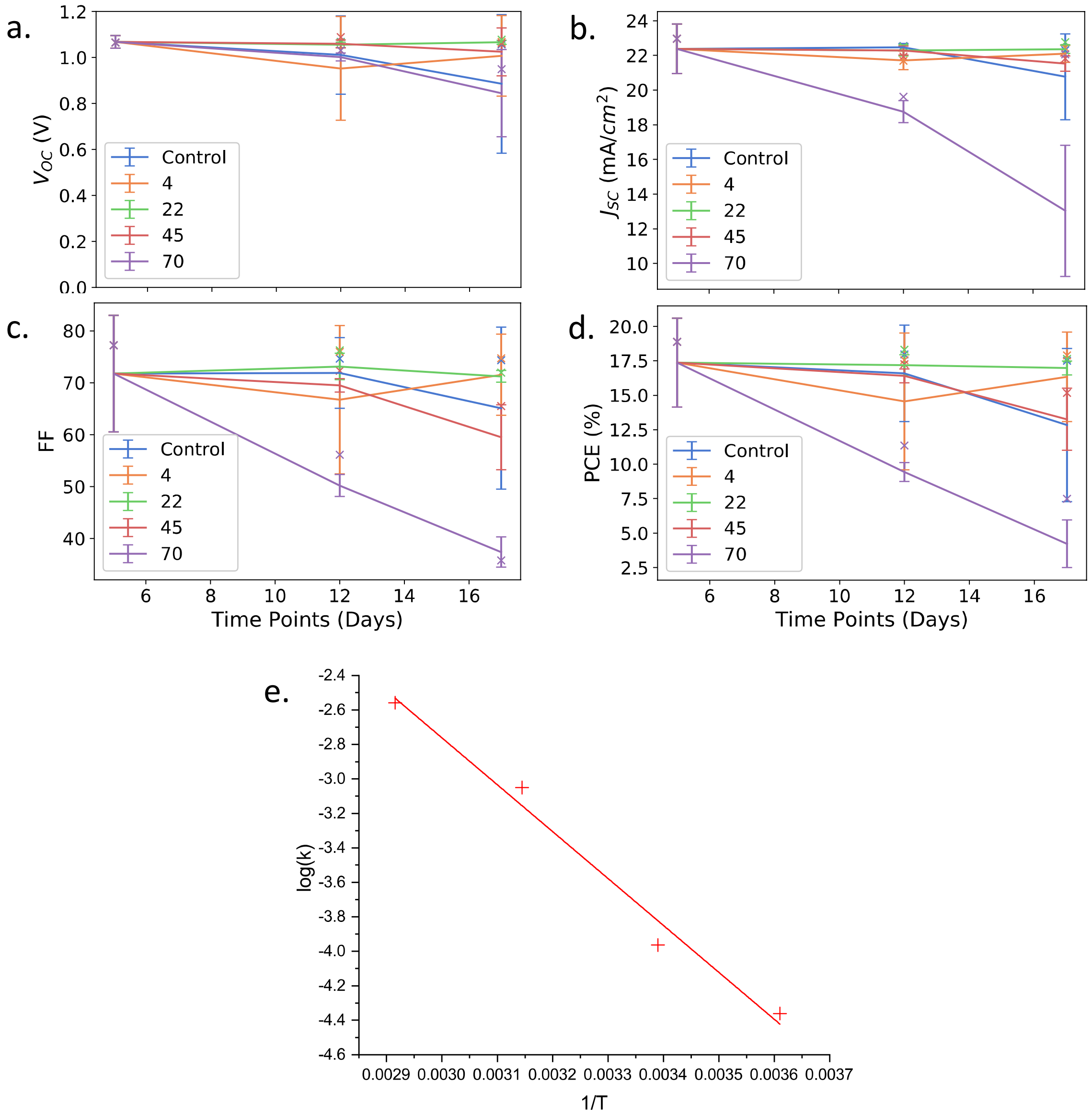

Figure S3 a-d. *Average and standard deviation of the performance metrics for devices made from a TC-mixed (DMF:DMSO 4:1) solution aged at various temperatures over a 3 week period. Part* ***e*** *shows a linear plot of log(λ/t) against 1/T (see equation 1) where k = λ/t represents rate of performance reduction per hour and T is temperature in K. Here a best fit to the data gives the activation energy* E_A/k_B.

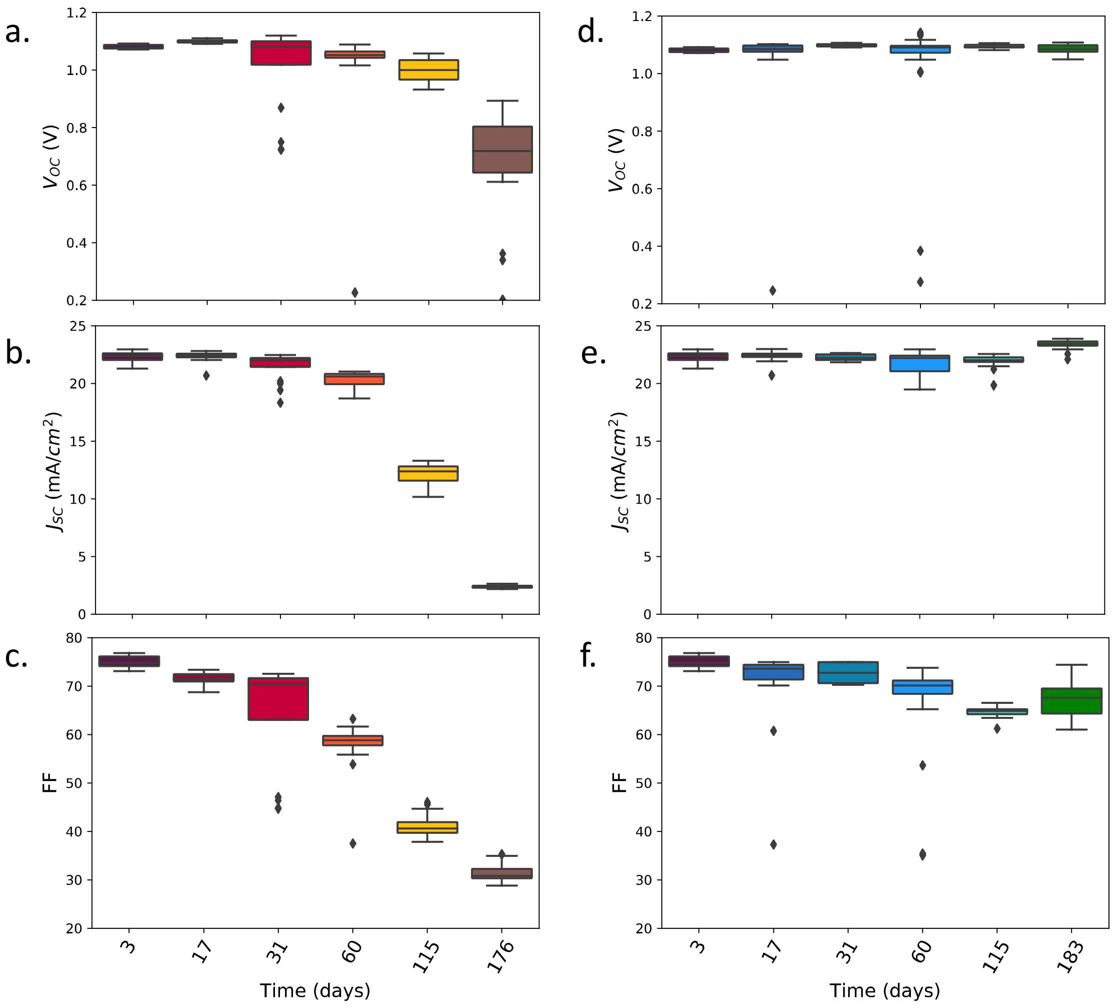

Figure S4. *V_{OC}, J_{SC}, and FF for devices made from RT-* ***(a-c)*** *and LT-aged* ***(d-f)*** *TC-mixed solutions. Results for the 183- and 176-day time point made from new, undisturbed precursor solutions that were aged under identical conditions to data points presented at the other time points.*

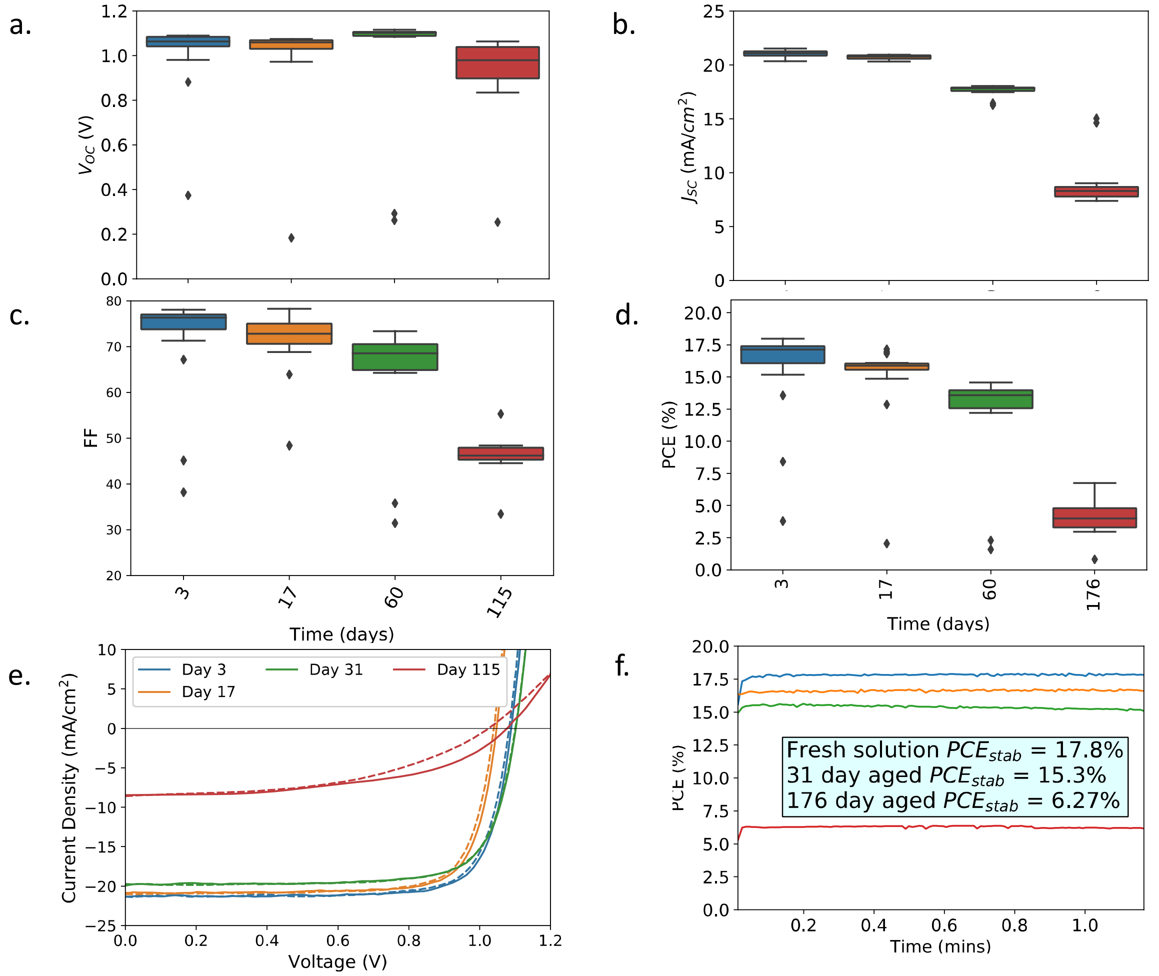

Figure S5. *RT-aged TC-DMSO device box-plots.* ***(a)*** V_{OC}, ***(b)*** J_{SC}, ***(c)*** *FF,* ***(d)*** *PCE,* ***(e)*** *J-V curves and* ***(f)*** *stabilised power output measurements for the best performing devices.*

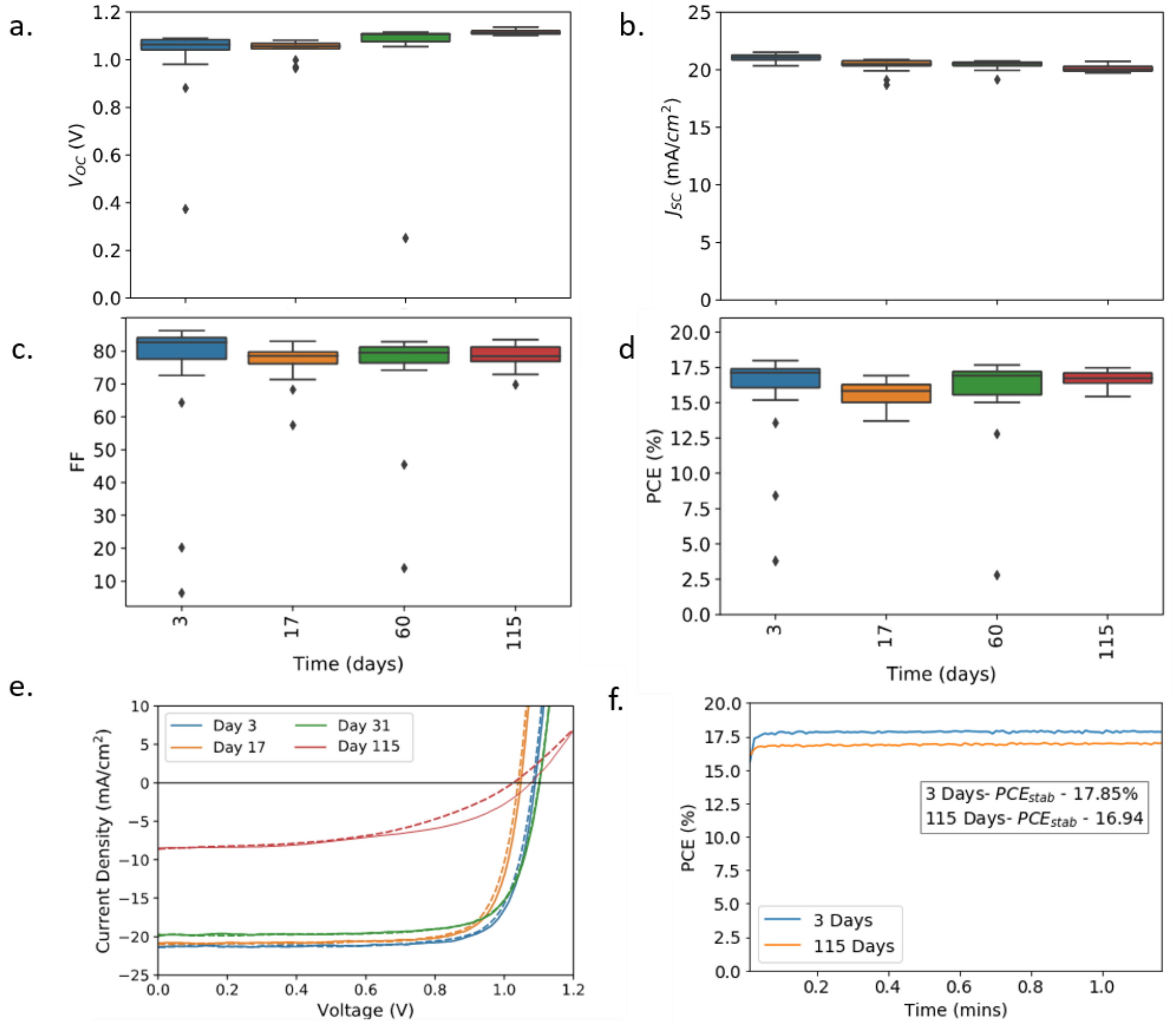

Figure S6. *LT-aged TC-DMSO device metrics.* ***(a)*** *V_{OC},* ***(b)*** *J_{SC},* ***(c)*** *FF,* ***(d)*** *PCE,* ***(e)*** *J-V curves and* ***(f)*** *stabilised power output measurements for the best performing devices.*

Storage time (Days)	V_{OC} (V)	J_{SC} (mA/cm^2)	FF	PCE (%)	Number of devices
Fresh	1.08 ± 0.01 (1.09)	22.3 ± 0.4 (22.7)	75.1 ± 1.1 (76.9)	18.1 ± 0.5 (19.0)	7
17	1.10 ± 0.06 (1.11)	22.3 ± 0.5 (22.7)	71.6 ± 1.2 (73.2)	17.6 ± 1.2 (18.5)	4
31	1.01 ± 0.14 (1.11)	21.5 ± 1.2 (22.3)	64.5 ± 10.9 (71.2)	14.6 ± 4.4 (17.9)	4
60	1.06 ± 0.02 (1.08)	20.4 ± 0.5 (20.6)	58.8 ± 2.0 (63.2)	12.7 ± 0.7 (14.2)	6
115	1.00 ± 0.04 (1.05)	12.1 ± 1.0 (12.9)	41.1 ± 2.2 (45.5)	5.0 ± 0.6 (6.1)	5

Table 2. *Device parameters: average (best) to accompany Figure 1a-c. Here the TC was dissolved in DMF/DMSO solvent and stored at RT. Devices were then made from these solutions at several time points as indicated.*

Storage time (Days)	V_{OC} (V)	J_{SC} (mA/cm^2)	FF	PCE (%)	Number of devices
Fresh	1.08 ± 0.01 (1.09)	22.3 ± 0.4 (22.7)	75.1 ± 1.1 (76.9)	18.1 ± 0.5 (19.0)	7
17	1.08 ± 0.02 (1.11)	22.5 ± 0.3 (22.6)	72.6 ± 3.3 (74.9)	17.7 ± 0.8 (18.5)	5
31	1.10 ± 0.01 (1.10)	22.3 ± 0.3 (22.7)	72.7 ± 2.1 (75.0)	17.8 ± 0.6 (18.6)	2
60	1.09 ± 0.03 (1.13)	21.8 ± 1.0 (22.7)	69.7 ± 4.1 (73.2)	16.6 ± 1.5 (18.9)	6
115	1.10 ± 0.01 (1.10)	22.1 ± 0.4 (22.1)	64.9 ± 0.7 (66.6)	15.7 ± 0.3 (16.1)	4

Table 3. *Device parameters: average (best) to accompany Figure 1d-f. Here the TC was dissolved in DMF/DMSO solvent and stored at LT. Devices were then made from these solutions at several time points as indicated.*

Storage time (Days)	V_{OC} (V)	J_{SC} (mA/cm^2)	FF	PCE (%)	Number of devices
Fresh	1.05 ± 0.04 (1.09)	20.7 ± 0.3 (21.3)	74.5 ± 5.8 (77.6)	16.6 ± 1.7 (18.0)	9
17	1.05 ± 0.03 (1.06)	20.7 ± 0.2 (20.9)	72.9 ± 3.4 (77.4)	15.8 ± 0.9 (17.1)	5
60	1.10 ± 0.01 (1.11)	17.8 ± 0.2 (17.9)	68.9 ± 3.1 (75.4)	13.5 ± 0.7 (14.6)	6

Table 4. *Device parameters: average (best) to accompany Figure S4. Here the TC dissolved in DMSO solvent and stored at RT. Devices were then made from these solutions at several time points as indicated.*

Storage time (Days)	V_{OC} (V)	J_{SC} (mA/cm^2)	FF	PCE (%)	Number of devices
Fresh	1.05 ± .04 (1.09)	20.7 ± 0.3 (21.3)	74.5 ± 5.8 (77.6)	16.6 ± 1.7 (18.0)	9
17	1.04 ± 0.04	21.1 ± 0.6	73.2 ± 3.3	15.5 ± 0.9	4

	(1.09)	(20.7)	(75.5)	(16.9)	
60	1.09 ± 0.03 (1.11)	20.3 ± 0.5 (20.8)	73.0 ± 5.1 (76.2)	16.2 ± 1.5 (17.7)	6
115	1.12 ± 0.01 (1.12)	20.1 ± 0.3 (20.3)	74.2 ± 1.8 (76.7)	16.7 ± 0.6 (17.5)	4

Table 5. *Device parameters: average (best) to accompany Figure S5. Here the TC dissolved in DMSO solvent and stored at LT. Devices were then made from these solutions at several time points as indicated.*

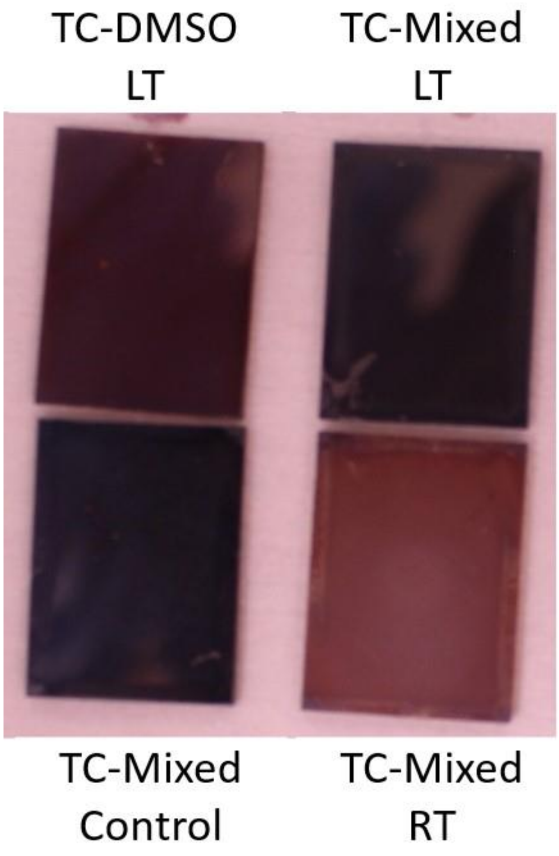

Figure S7. *Images of all films prepared for solutions aged for 4 months together with a control film prepared from a freshly prepared solution.*

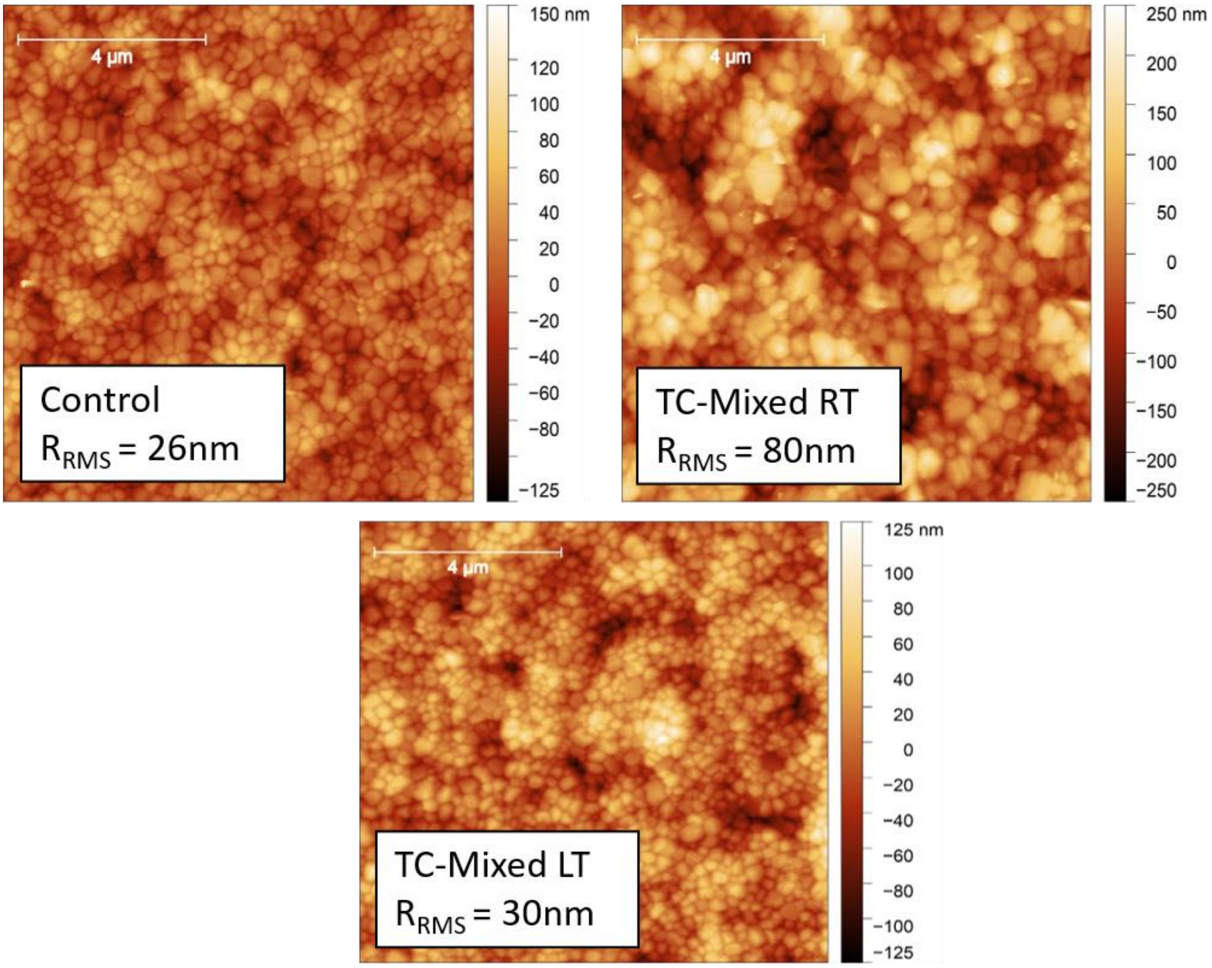

Figure S8. *AFM height images for films prepared from 115 day aged (LT and RT) TC-mixed precursor solutions, together with a control film prepared from a freshly prepared solution.*

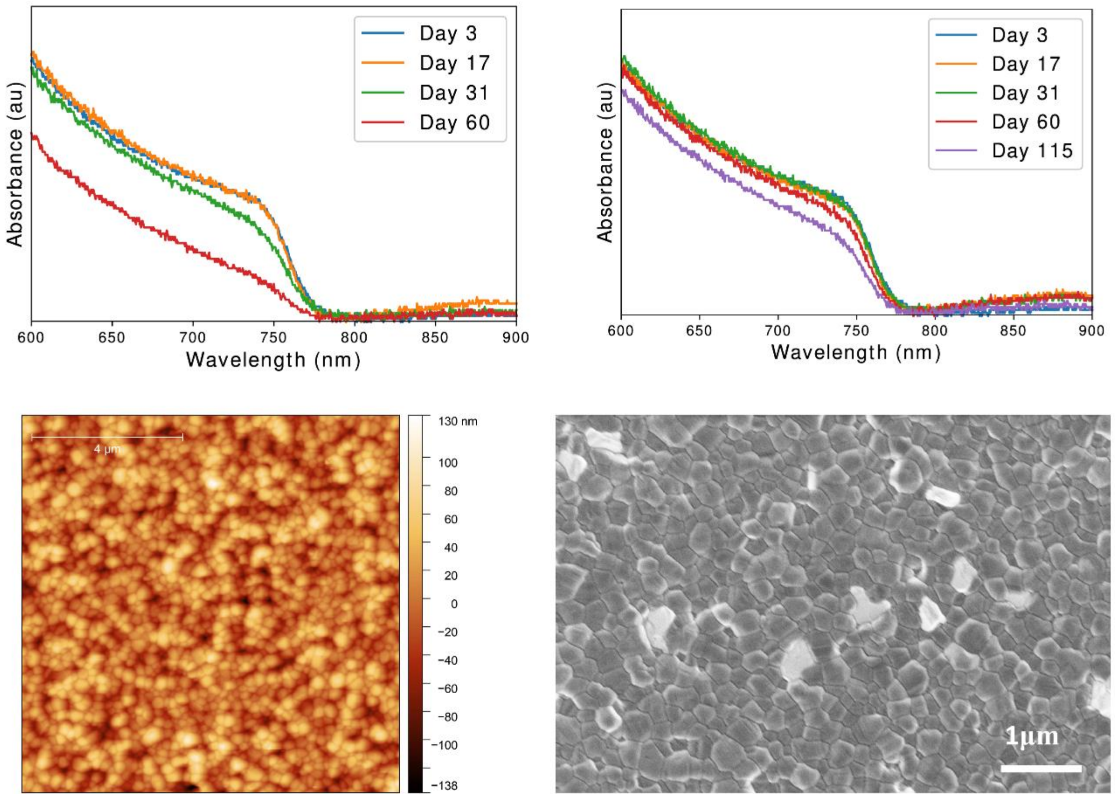

Figure S9. *UV-Vis absorption recorded from films made from **(a)** RT-aged and **(b)** LT-aged TC-DMSO solutions at several time points. **(c)** AFM and **(d)** SEM images of films made from 4 month RT-aged DMSO precursor solutions.*

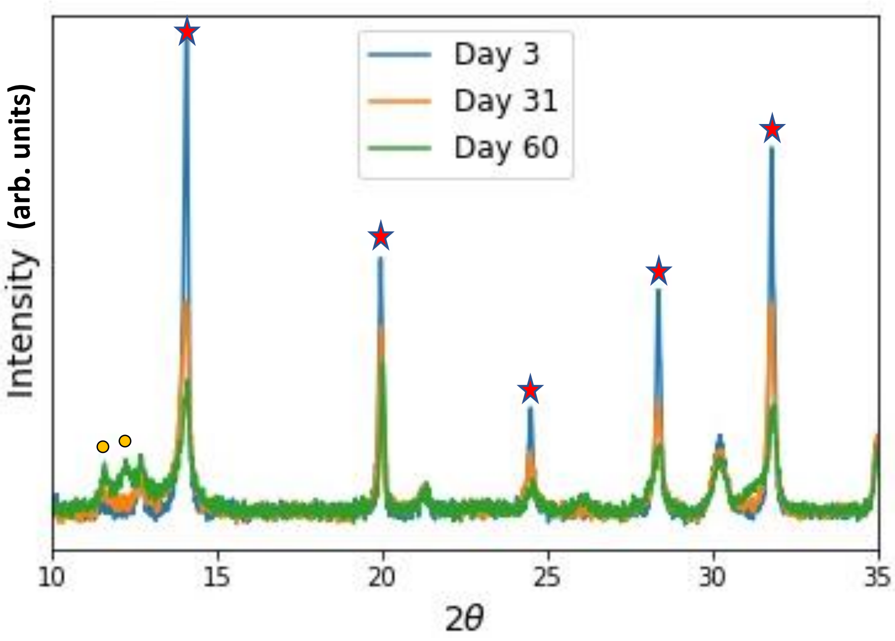

Figure S10. *XRD patterns recorded from a film prepared from a RT-aged TC-DMSO precursor solution aged for several time points over a 2 month period.*

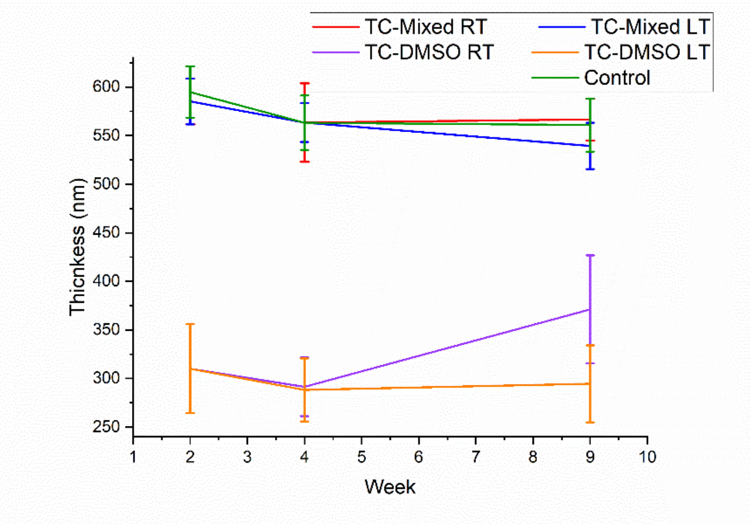

Figure S11. *Thicknesses of films prepared at various points in time from progressively aged precursor solutions. The composition of the precursor is shown in the figure legend. Thickness was measured at various points across the film using surface profilometry.*

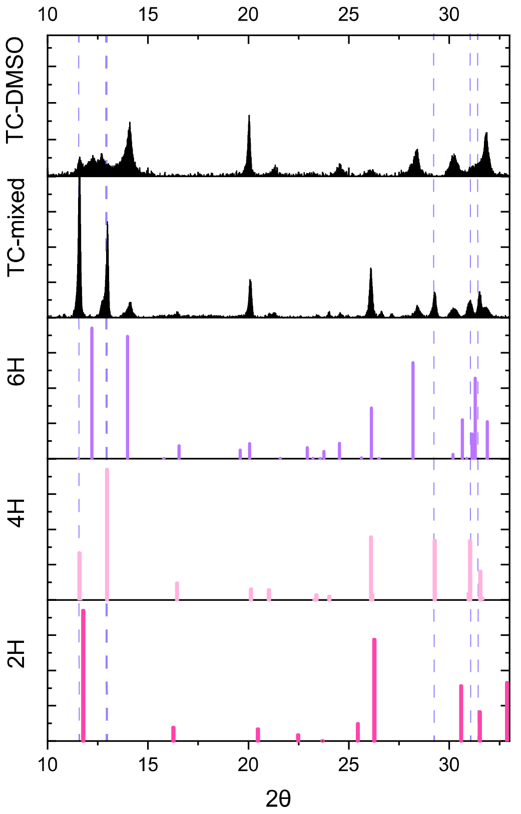

Figure S12. *Simulated data for different FAPbI$_3$ 2H,4H and 6H polytypes – based on CIF files from [2] - compared with XRD data from films created from 115-day RT-aged TC-mixed and TC-DMSO solutions. The dashed lines are used to guide the eye. The figure highlights the fact that different polytypes form in the films as the solutions age, and the nature of the polytype is apparently dependent on solvents. We find that the DMF/DMSO film exhibits many peaks that clearly align with a 4H polytype, (principally the peaks forming at 29.2° and 31.5°) indicating a combination of corner-sharing and face-sharing Pb-halide octahedra.*

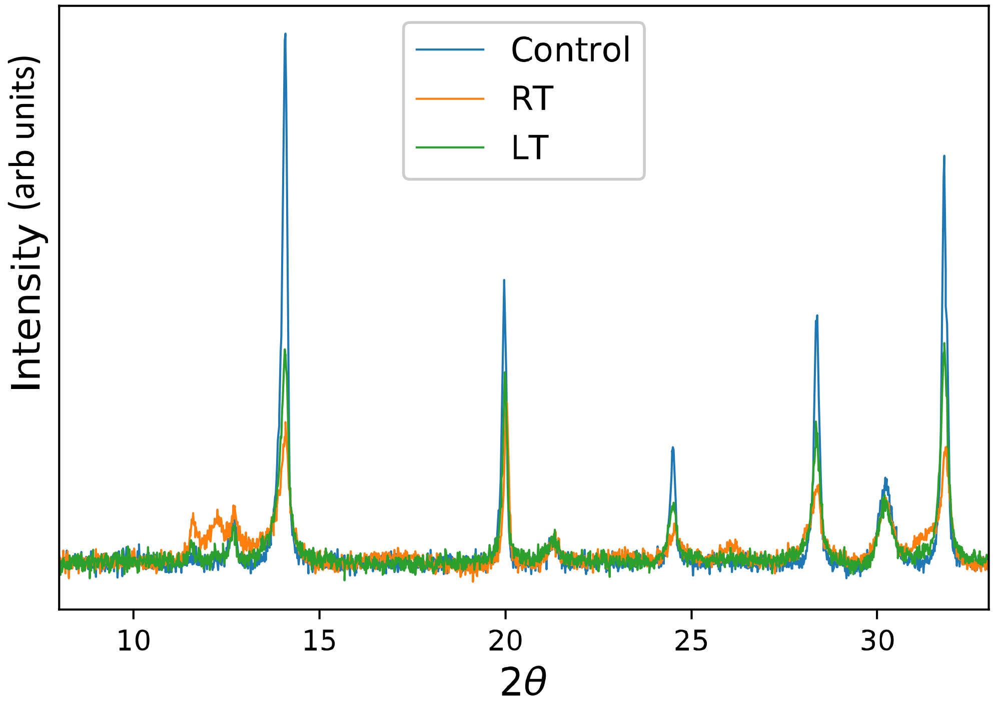

Figure S13. *XRD patterns for films made from 115-day-aged TC-DMSO solution stored at RT (orange) and LT (green) compared with a control made from a fresh DMSO solution.*

NMR

Peak positions were calibrated with respect to an internal isolated standard of 1,2,4,5-Tetrachloro-3-nitrobenzene (TCNB) (peak at 8.4364 ppm) in DMSO-d6 in a capillary tube which was placed inside the sample tube containing the precursor solution being measured. The spectra presented below therefore contain peaks that derive from both the sample and the standard. We highlight peaks associated with the TCNB standard using a blue box.

Note that the standard capillary tube used was washed before use with isopropanol and water. These species inside the capillary can be seen as contamination peaks at 4.3 ppm, 3.8 ppm, 1.0 ppm (for IPA) and 3.3 ppm (for water) in the following ^{1}H spectra. For the ^{1}H measurements, we find a chemical shift between the samples that are dissolved in different solvents.

It is important to emphasize that the exact chemical shift of any proton environment is highly dependent on its surroundings and can vary due to the due to concentration, pH or solvent co-ordination. Indeed, in all solutions (even though such solvents are deuterated), we observe some residual non-deuterated solvent (i.e. DMSO or DMF) that is generated through the replacement of deuterium by a proton. As there is a different chemical environment in the TC solution and TCNB solutions, we observe some shift of the peaks associated with the residual solvents; for example we see peaks at 2.2 and 2.5 ppm which are both associated with DMSO.

Deuterium Spectra

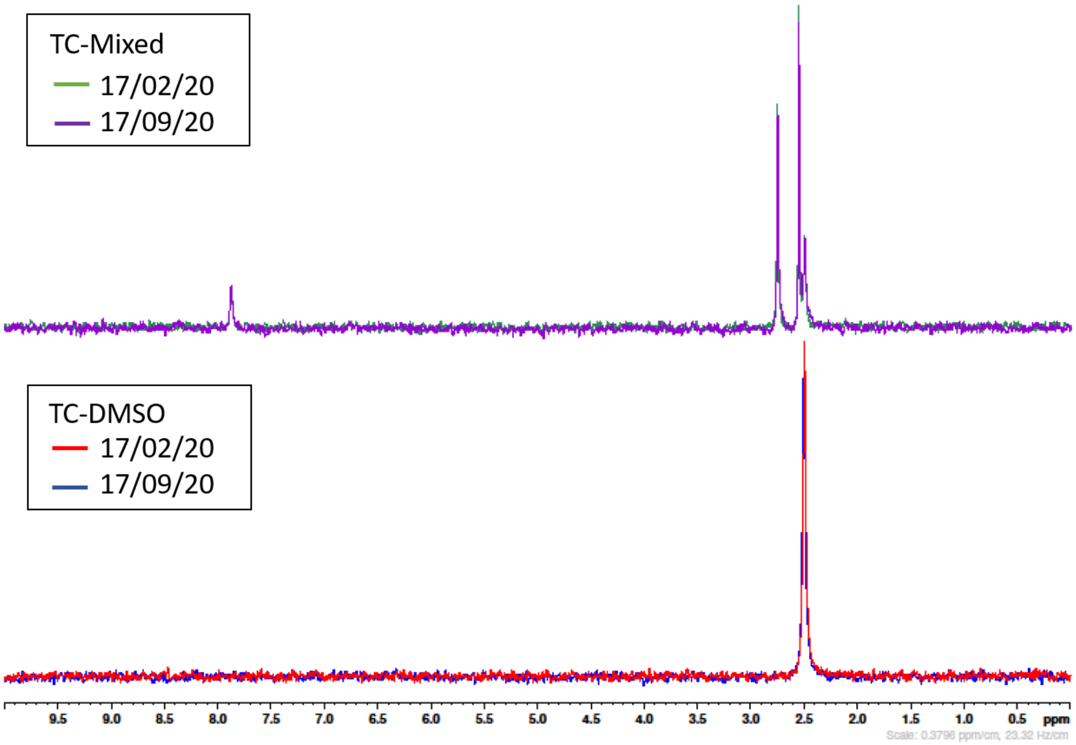

Figure S14. *^{2}H spectra of TC solutions dissolved in d_7-DMF /d_6-DMSO (4:1) (above) and d_6-DMSO (below) before and after 6 months storage at RT under nitrogen. Peaks at 2.5 ppm are assigned to d_6-DMSO and those at 2.55 ppm, 2.75 ppm and 7.87 ppm are assigned to d_7-DMF.*

TC solution in d_7-DMF/d6-DMSO

Peaks at 7.60 ppm and 2.29ppm are assigned as residual DMF impurities, and 2.24 ppm peak is assigned to residual DMSO impurities, confirmed with ^{2}H NMR spectra on similar solutions. Peaks highlighted in blue are from the internal standard.

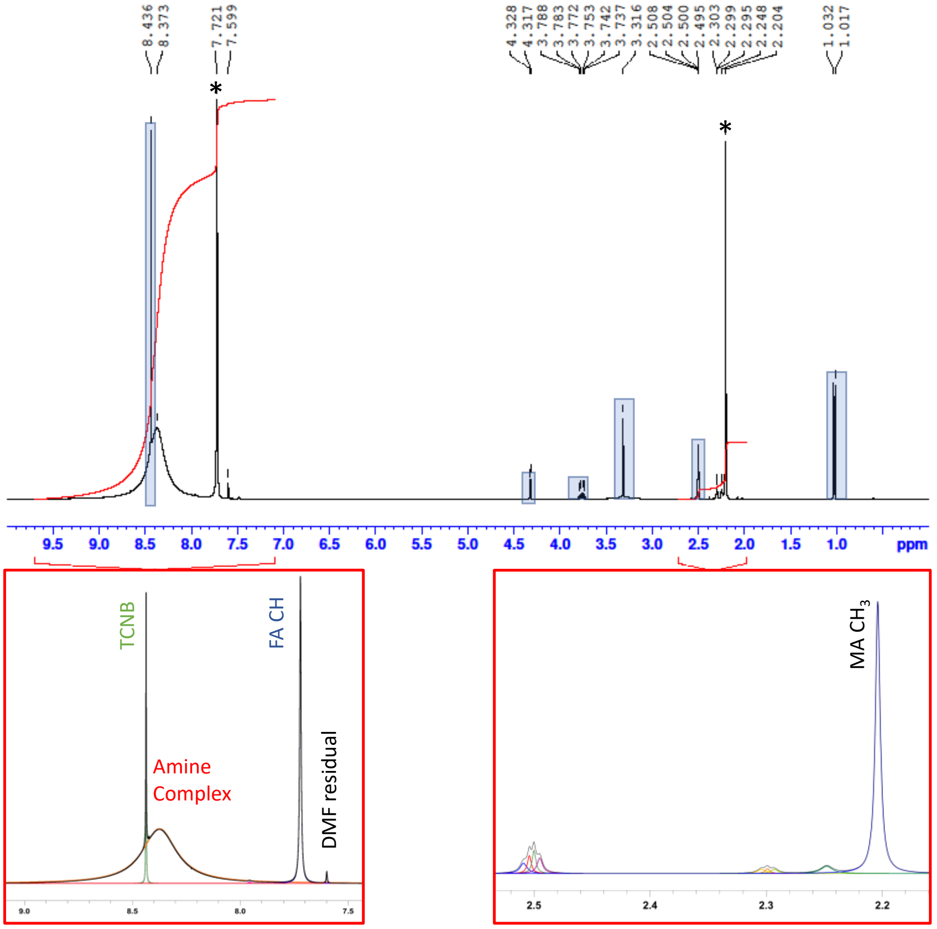

Figure S15. *Full ^{1}H NMR spectrum of TC precursor in d_7-DMF/d_6-DMSO (4:1) after 7 days aging. Asterisks denote peaks of interest: Peaks at 7.72 ppm and 2.20 ppm are assigned to CH of FA and CH_3 of MA respectively. For completeness, the deconvoluted spectra are also shown in the bottom two panels to show how the area of overlapping peaks are calculated (with our methods following the protocol outlined in ref [3]). We tabulate the area of integrated regions as extracted from the spectra in the in Table 6.*

Peak	Integral Area	Concentration (mM)	# of Hydrogens
TCNB (Internal Standard)	1.00	383	1
Amine Complex	19.08	7307	N/A
FA CH	4.05	1550	1
MA CH_3	2.51	961	3

Table 6*. Integral areas and subsequent concentrations for the integrals shown in Figure S15.*

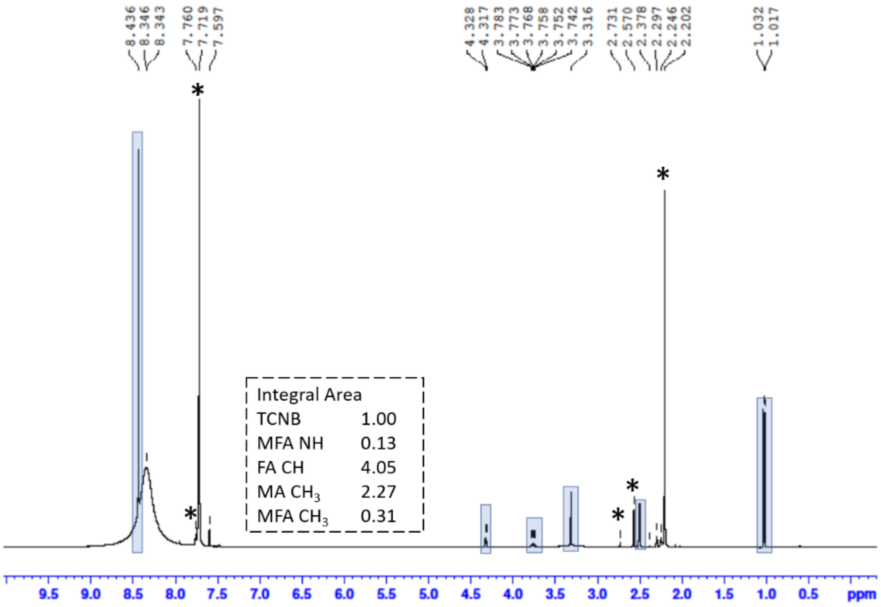

Figure S16. *Full* [1]*H NMR spectrum of TC precursor in* d_7*-DMF/*d_6*-DMSO (4:1) after 14 days aging. Asterisks denote peaks of interest: Peaks at 7.72 ppm and 2.20 ppm are assigned to the CH of FA and* CH_3 *of MA respectively. Emerging peaks at 2.57 ppm and 7.76 ppm are assigned to* CH_3 *and NH of MFA respectively, with the peak at 2.73 ppm assigned to the* CH_3 *of DMFA. Integral values, referenced to the TCNB standard as 1.00, are shown in the inset box.*

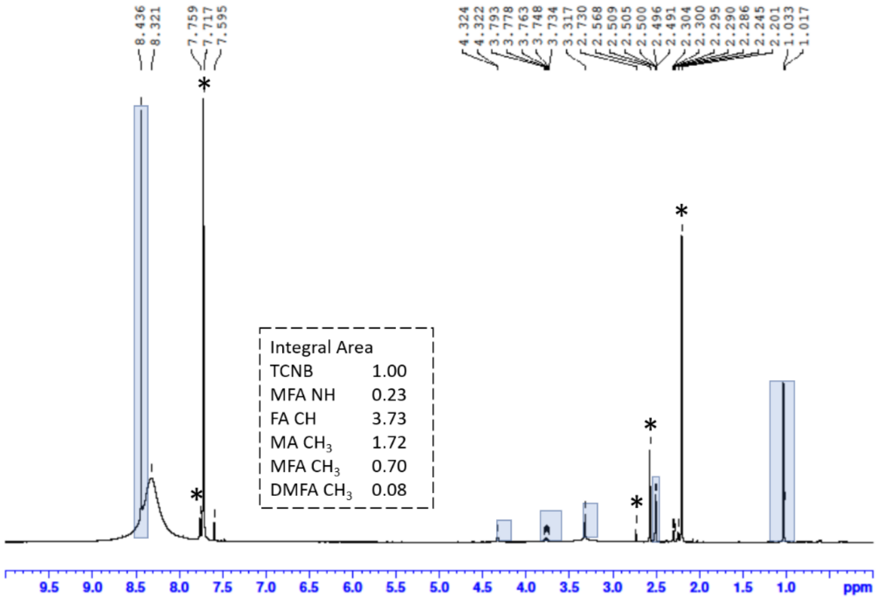

Figure S17. *Full* [1]*H NMR spectrum of TC precursor in* d_7*-DMF/*d_6*-DMSO (4:1) after 21 days aging. Asterisks denote peaks of interest: Peaks at 7.72 ppm and 2.20 ppm are assigned to the CH of FA and* CH_3 *of MA respectively. Emerging peaks at 2.57 ppm and 7.76 ppm are assigned to* CH_3 *and NH of MFA respectively, with the peak at 2.73 assigned to the* CH_3 *of DMFA. Integral values, referenced to the TCNB standard as 1.00, are shown in the inset box.*

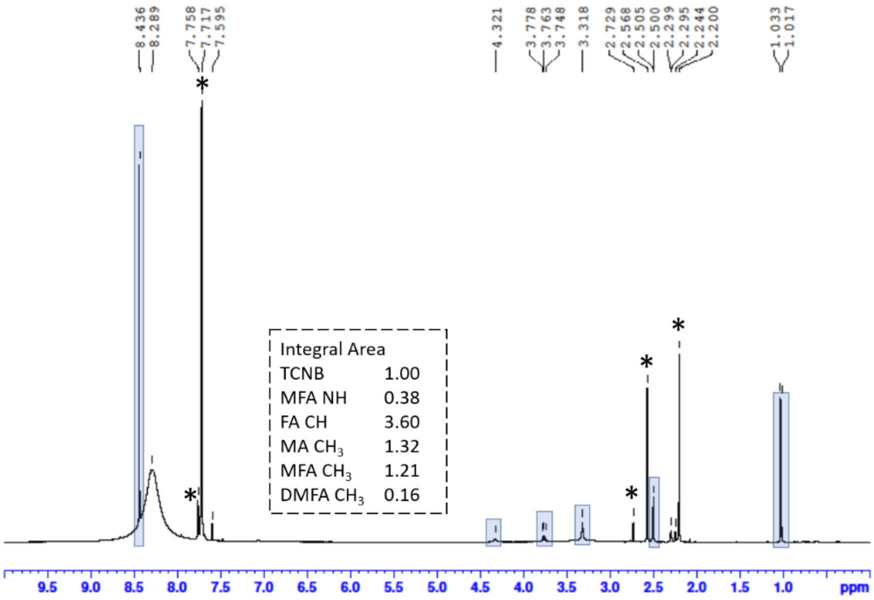

Figure S18. *Full ^{1}H NMR spectrum of TC precursor in d_7-DMF/d_6-DMSO (4:1) after 28 days aging. Asterisks denote peaks of interest: Peaks at 7.72 ppm and 2.20 ppm are assigned to the CH of FA and CH_3 of MA respectively. Emerging peaks at 2.57 ppm and 7.76 ppm are assigned to CH_3 and NH of MFA respectively, with the peak at 2.73 assigned to the CH_3 of DMFA Integral values, referenced to the TCNB standard as 1.00, are shown in the inset box.*

TC solution in d_6-DMSO

The peak at 2.22 ppm is assigned to residual DMSO impurities, confirmed with ^{2}H NMR spectra on similar solutions.

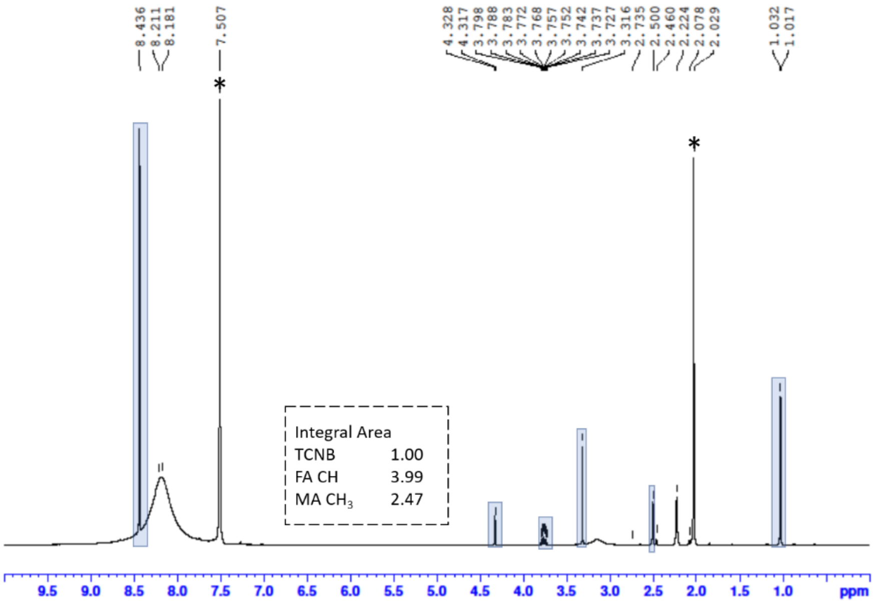

Figure S19. *Full ^{1}H NMR spectrum of TC precursor in d_6-DMSO after 7 days aging. Asterisks denote peaks of interest: Peaks at 7.51 ppm and 2.02 ppm are assigned to the CH of FA and CH_3 of MA respectively. Integral values, referenced to the TCNB standard as 1.00, are shown in the inset box.*

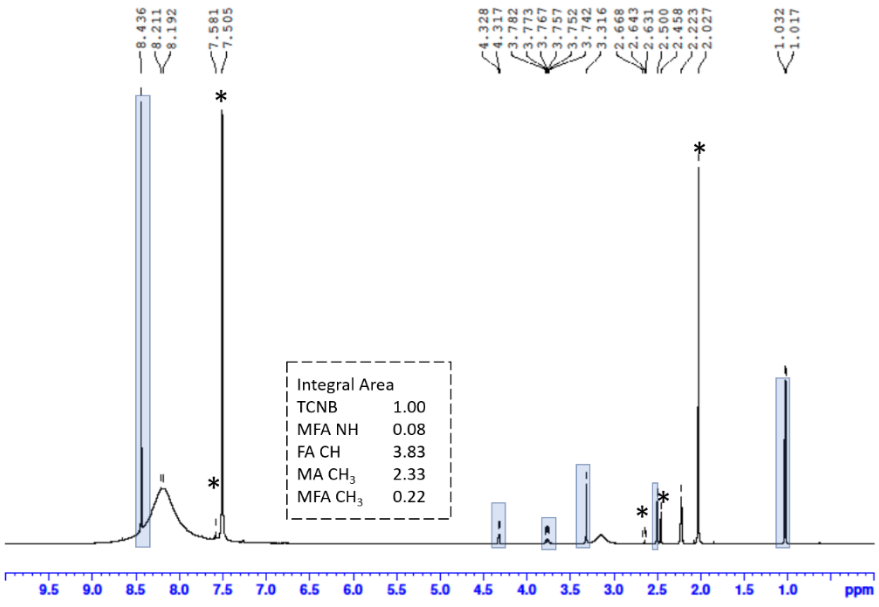

Figure S20. *Full ^{1}H NMR spectrum of TC precursor in d_6-DMSO after 14 days aging. Asterisks denote peaks of interest: Peaks at 7.51 ppm and 2.02 ppm are assigned to the CH of FA and CH_3 of MA respectively. Emerging peaks at 2.46 ppm and 7.58 ppm are assigned to CH_3 and NH of MFA respectively, with the peak at 2.67 assigned to the CH_3 of DMFA. Integral values, referenced to the TCNB standard as 1.00, are shown in the inset box.*

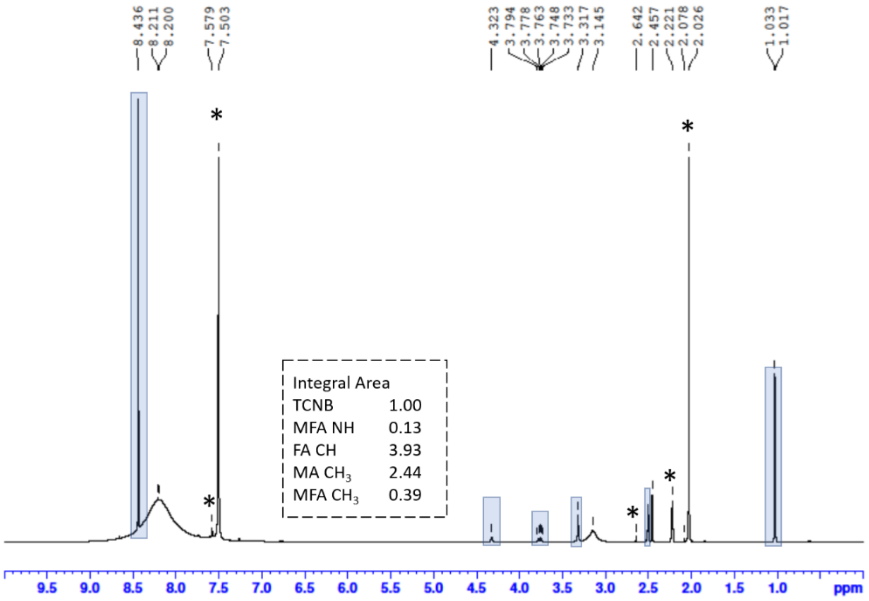

Figure S21. *Full ^{1}H NMR spectrum of TC precursor in d_6-DMSO after 21 days aging. Asterisks denote peaks of interest: Peaks at 7.51 ppm and 2.02 ppm are assigned to the CH of FA and CH_3 of MA respectively. Emerging peaks at 2.46 ppm and 7.58 ppm are assigned to CH_3 and NH of MFA respectively, with the peak at 2.67 ppm assigned to the CH_3 of DMFA. Integral values, referenced to the TCNB standard as 1.00, are shown in the inset box.*

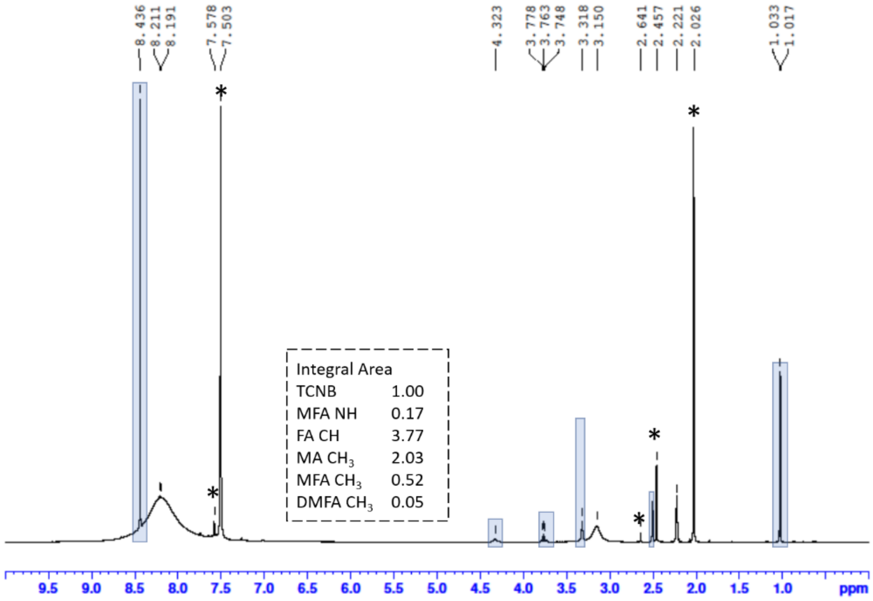

Figure S22. *Full ^{1}H NMR spectrum of TC precursor in d_6-DMSO after 28 days aging. Asterisks denote peaks of interest: Peaks at 7.51 ppm and 2.02 ppm are assigned to the CH of FA and CH_3 of MA respectively. Emerging peaks at 2.46 ppm and 7.58 ppm are assigned to CH_3 and NH of MFA respectively, with the peak at 2.67 ppm assigned to the CH_3 of DMFA. Integral values, referenced to the TCNB standard as 1.00, are shown in the inset box.*

TC solutions in d_7-DMF

Peaks at 7.60 ppm and 2.29 ppm are assigned to residual DMF impurities confirmed with ^{2}H NMR spectra on similar solutions.

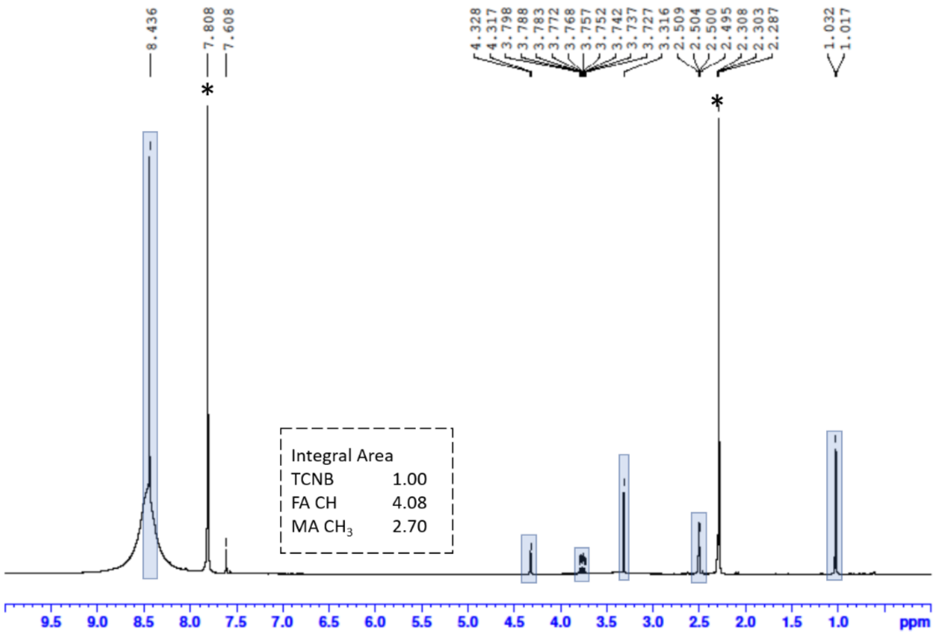

Figure S23. *Full ^{1}H NMR spectrum of TC precursor in d_7-DMF after 7 days aging. Asterisks denote peaks of interest: Peaks at 7.81 ppm and 2.28 ppm are assigned to the CH of FA and CH_3 of MA respectively. Integral values, referenced to the TCNB standard as 1.00, are shown in the inset box.*

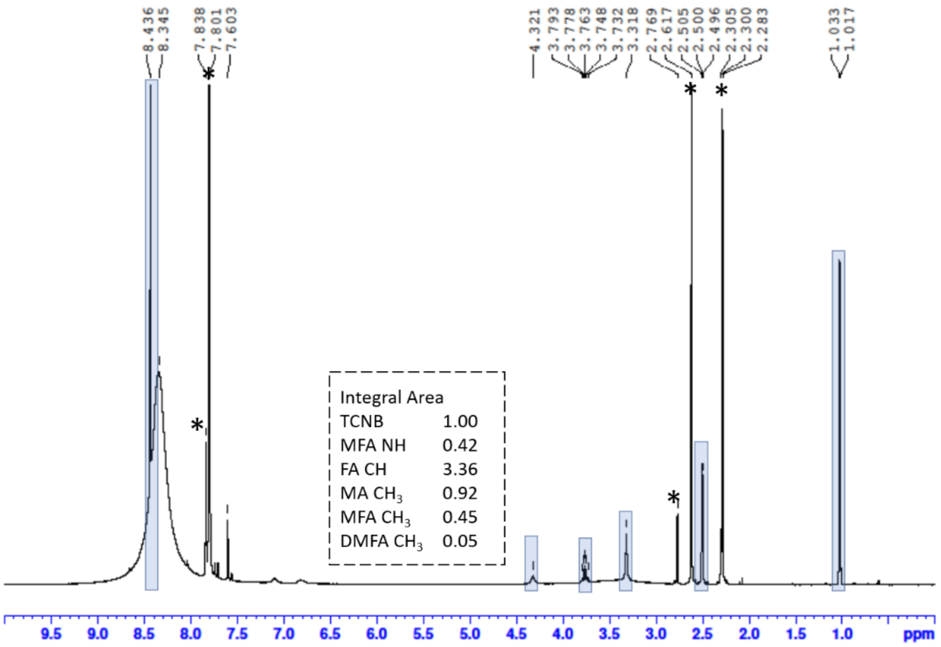

Figure S24. *Full ^{1}H NMR spectrum of TC precursor in d_7-DMF after 28 days aging. Asterisks denote peaks of interest: Peaks at 7.80 ppm and 2.9 ppm are assigned to the CH of FA and CH_3 of MA respectively. Emerging peaks at 2.61 ppm and 7.84 ppm are assigned to CH_3 and NH of MFA respectively, with the peak at 2.77 assigned to the CH_3 of DMFA. Integral values, referenced to the TCNB standard as 1.00, are shown in the inset box.*

Estimated Molarities

Aging (days)	MA CH_3 (2.20 ppm)	FA CH (7.72 ppm)	MFA NH (7.76 ppm)	MFA CH_3 (2.57 ppm)	DMFA CH_3 (2.73 ppm)
7	320	1550			
14	290	1550	50	40	
21	219	1430	90	90	10
28	169	1380	145	155	20

Table 7. Molarity (in mM) of organic molecules within solution estimated from NMR measurements (within ±10%) of aging TC solutions in d_7-DMF/d_6-DMSO. This was measured using Topspin and referenced to the internal standard TCNB having a molarity of 383mM. Peaks consisting of multiple hydrogen signals have been scaled to reflect their true concentration.

Aging (Days)	MA CH_3 (2.0p pm)	FA NH (7.5 ppm)	MFA NH (7.6 ppm)	MFA CH_3 (2.5 ppm)	DMFA CH_3 (2.6 ppm)
7	315	1527	0	0	0
14	298	1465	29	28	
21	311	1506	51	50	
28	260	1445	66	66	7

Table 8. Molarity (in mM) of organic molecules within solution estimated from NMR measurements (within ±10%) of aging TC solutions in d_6-DMSO. This was measured using Topspin and referenced

to the internal standard TCNB having a molarity of 383mM. Peaks consisting of multiple hydrogen signals have been scaled to reflect their true concentration.

Aging (days)	MA CH_3 (2.30 ppm)	FA CH (7.80 ppm)	MFA NH (7.83 ppm)	MFA CH_3 (2.61 ppm)	DMFA CH_3 (2.77 ppm)
7	345.33	1561.329			
28	117.6	1287.364	161.12	173.6	20.5

Table 9. *Molarity (in mM) of organic molecules within solution estimated from NMR measurements (within ±10%) of aging TC solutions in d_7-DMF. This was measured using Topspin and referenced to the internal standard TCNB having a molarity of 383mM. Peaks consisting of multiple hydrogen signals have been scaled to reflect their true concentration.*

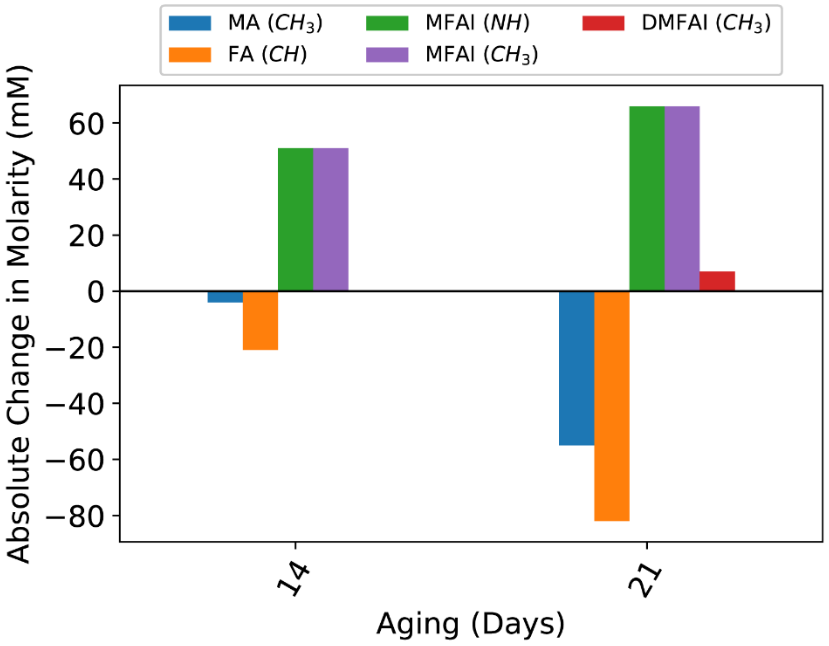

Figure S25. *TC- d_6-DMSO precursor solution absolute molarity changes calculated from NMR data. Comparing to data shown in Figure 5 of the main paper, there is a much smaller molarity change than occurs in comparably aged DMF/DMSO precursors.*

References

[1] C. Avenel, O. Raccurt, J.-L. Gardette, S. Therias, *npj Mater. Degrad.* **2019**, *3*, 27.

[2] P. Gratia, I. Zimmermann, P. Schouwink, J.-H. Yum, J.-N. Audinot, K. Sivula, T. Wirtz, M. K. Nazeeruddin, *ACS Energy Lett.* **2017**, *2*, 2686–2693.

[3] M. Lipfert, M. K. Rout, M. Berjanskii, D. S. Wishart, in *NMR-Based Metabolomics Methods Protoc.* (Eds.: G.A.N. Gowda, D. Raftery), Springer New York, New York, NY, **2019**, pp. 429–449.
